# Reviewing the Role of the Endocannabinoid System in the Pathophysiology of Depression

**DOI:** 10.3389/fphar.2021.762738

**Published:** 2021-12-06

**Authors:** Ines Gallego-Landin, Alba García-Baos, Adriana Castro-Zavala, Olga Valverde

**Affiliations:** ^1^ Neurobiology of Behaviour Research Group (GReNeC—NeuroBio), Department of Experimental and Health Sciences, Universitat Pompeu Fabra, Barcelona, Spain; ^2^ Neuroscience Research Programme, IMIM-Hospital del Mar Research Institute, Barcelona, Spain

**Keywords:** endocannabinoid system, HPA-axis, major depressive disorder, neurogenesis, neuroinflammation

## Abstract

Major depressive disorder is a high-impact, debilitating disease and it is currently considered the most prevalent mental illness. It is associated with disability, as well as increased morbidity and mortality. Despite its significant repercussions in our society, its exact pathophysiology remains unclear and therefore, available antidepressant treatment options are limited and, in some cases, ineffective. In the past years, research has focused on the development of a multifactorial theory of depression. Simultaneously, evidence supporting the role of the endocannabinoid system in the neurobiology of neuropsychiatric diseases has emerged. Studies have shown that the endocannabinoid system strongly impacts neurotransmission, and the neuroendocrine and neuroimmune systems, which are known to be dysfunctional in depressive patients. Accordingly, common antidepressants were shown to have a direct impact on the expression of cannabinoid receptors throughout the brain. Therefore, the relationship between the endocannabinoid system and major depressive disorder is worth consideration. Nevertheless, most studies focus on smaller pieces of what is undoubtedly a larger mosaic of interdependent processes. Therefore, the present review summarizes the existing literature regarding the role of the endocannabinoid system in depression aiming to integrate this information into a holistic picture for a better understanding of the relationship between the two.

## Introduction

Major Depressive Disorder (MDD) is the most common mood disorder characterized by the loss of interest or pleasure in activities, unjustified feelings of worthlessness and presence of depressed mood or aversion to activity (American Psychiatric Association, 2013).

According to the World Health Organization, each year, 25% of the European population suffers from anxiety or depressive disorders (World Health Organization, 2020). Its prevalence has increased by 18% between 2005 and 2015 (Akil et al., 2018) and currently, it constitutes the leading cause of disability. It is calculated that around 80% of MDD patients suffer from some sort of impairment during their daily life (Pratt and Brody, 2008). Importantly, this disorder occurs chronically throughout the lifespan: half of the patients undergoing depressive episodes continue to experience them with increasing frequency and severity over time. Furthermore, untreated MDD is the leading cause of suicide (National Collaborating Centre for Mental Health (UK), 2010) which, simultaneously is the second leading cause of premature death among 15–29 year-old individuals and number three among the 15–44 age group (Bachmann, 2018). On this basis, the impact of MDD in society should not be dismissed.

Currently, the most common treatment for MDD involves administration of antidepressant medications combined with psychiatric or psychological treatment. Although these medications do have positive effects, less than 50% of the patients accomplish full remission after the first pharmacological treatment (Trivedi et al., 2006).

Furthermore, the large-scale societal effects of MDD are highlighted by the responses to global coronavirus disease 2019 (COVID-19). Notably, due to the imposed lockdown, epidemiological studies show an increase in the percentage of people reporting mood disorders (Benke et al., 2020; Morin et al., 2020). Between August 2020 and February 2021, the number of individuals with recent anxiety or depressed symptoms grew by 5% (from 36.4 to 41.5%) (Vahratian et al., 2021). Therefore, the relevance of MDD-related studies is, arguably, more important than ever.

Unfortunately, although many risk factors have been identified to contribute to the development of MDD, the current hypothesized etiologies fail to explain its underlying mechanisms due to the complex interplay of the risk factors and the individual differences in symptomatology. For instance, social, genetic, hormonal or lifestyle factors all play a role and contribute to a higher risk for MDD.

Recently, the field of the endocannabinoids has grown in popularity since the discovery of endogenous cannabinoid receptors in the brain (Matsuda et al., 1990) and their most relevant endocannabinoids ligands. Multiple studies have reported its role on a variety of brain structures and cognitive functions like memory, appetite, metabolism, immune system, mood and sleep (Murillo-Rodriguez et al., 2011; Tanasescu and Constantinescu, 2010; Zanettini et al., 2011).

Although this field of research is relatively novel, there exist millennia-old reports about the use of the Cannabis Sativa plant as a medicinal herb to treat conditions like anxiety and mania (Zuardi, 2006). Currently, in the United States, a cross-sectional survey revealed that depression is the third reported condition (50.3% of users) for usage of therapeutic cannabis (Sexton et al., 2016), and users reported a 86% reduction in symptomatology (Sexton et al., 2016).

In accordance with the increase of anxiety and depressive symptoms during the COVID-19 pandemic, a recent study reported increased cannabis consumption in those who declared usage to cope with depressive symptoms (Bartelet al., 2020). Similarly, during the lockdown, more users increased than decreased cannabis consumption regarding both, frequency and quantity (Laar et al., 2020). This is in line with the observation that cannabis users increase use during times of elevated stress (Kaplan et al., 1986).

On this basis, a potential role for the endocannabinoid system (ECS) as a contributor to the pathophysiology of MDD has been explored (Patel and Hillard, 2009). In fact, ongoing clinical studies are already investigating cannabinoid-based medications as treatment options for MDD (Sarris et al., 2020). Nevertheless, evidence on the use of these substances is scarce and future research is needed.

The present review aims to provide a concise overview of the existing knowledge on the ECS and its influence on the development of MDD. First, a summary of the most relevant and recent findings regarding both phenomena will be presented. Second, and more specific, the article will highlight the potential of the ECS as a unionizing figure between the currently proposed models for the pathophysiology of MDD, focusing on the most recent empirical evidence associating both phenomena as well as the existing knowledge gaps that should be addressed by future research.

## Major Depressive Disorder

MDD is one of the most common mood disorders worldwide. Although the prevalence percentage varies among reports, it is estimated that 3.4–4.4% of the global population suffers from MDD, which translates to 264–322 million people worldwide (World Health Organization, 2017; Dattani et al., 2018). It has been observed to be twice as common in females (5.1%) than in males (3.6%), although the fundamental cause of this gender gap has not been identified. Several impact factors have been proposed, such as: biological differences as well as socioeconomic factors like discrimination and poverty (Belle and Doucet, 2003; Rai et al., 2013). In particular, females also suffer specific forms of MDD like postpartum depression and postmenopausal depression and anxiety. These are associated with alterations in the fluctuation of ovarian hormones, which might directly or indirectly contribute to the elevated prevalence (Albert, 2015). Nonetheless, the underlying mechanisms are unclear and female-specific treatments have not yet been developed.

The most prescribed treatment for MDD is the administration of common antidepressants. Among them, Selective Serotonin Reuptake Inhibitors (SSRIs) are the most widely prescribed (64.2% of US patients in 2015), followed by Serotonin and Norepinephrine Reuptake Inhibitors (SNRIs) (16.4% of patients) (Luo et al., 2020). In fact, approximately 10–35% of patients do not remit from MDD even after several treatment attempts (Nemeroff, 2007; Kubitz et al., 2013).

This lack of availability of reliable medication for such a high-impact, debilitating disease is, in part, due to the inadequacy of current hypothesized etiologies to explain its underlying mechanisms. The above-mentioned available medications are a result of a serendipitous discovery of the antidepressant effects of monoamine oxidase inhibitor (MOAIs) Iproniazid during the 1950’s, which was used at the time against tuberculosis (López-Muñoz and Alamo 2009). Furthermore, also during this decade, imipramine, the first identified tricyclic antidepressant (TCAs), was synthesized for the first time as an antihistaminic medication (López-Muñoz and Alamo 2009). Initially, although academic psychiatrists of the time regarded these drugs as cures for certain severe depressive states, the mechanisms by which these were exerted remained unknown. The first clue arrived with the observation that both classes of antidepressants increased catecholamine levels in the brain via distinct biochemical pathways (Spectoret al., 1960). This leads to the development of the monoamine theory, which inaugurated the modern psychopharmacological era in psychiatry. Shortly, the monoamine theory hypothesized that, since the enhancement of monoamine concentrations in the brain had antidepressant properties, depression itself was a consequence of depletion of centrally available monoamines. This generated a debate over the importance of different monoamines in the etiology of MDD. Two years later, serotonin (5-HT) deficiency was linked to the development of MDD (Coppen, 1967). Thus, after these years, there were two competing monoamine theories which were held for decades, to later evolve into monoamine receptor theories, associating MDD with the alteration of this neurotransmission (Stahl, 1984).

This way, the monoamine hypothesis of MDD dominated our understanding of the pathophysiology of depression and the action of the available antidepressants. Overall, it is reasonable to hypothesize that depressive symptoms are a result of inadequate monoamine neurotransmission. In fact, the best pharmacological treatments for depression, to date, compromise drugs that enhance monoaminergic levels (SSRIs, SNRIs, etc.). Nonetheless, in more recent years, serious limitations were encountered for this theory, which lead to the speculation that factors beyond monoamine imbalance and deficiency must also be involved.

One of the largest challenges in this matter is accounting for the extensive constellation of symptoms exhibited by depressed patients. The Diagnostic Statistical Manual 5 (DSM-5) requires the presence of 5 out of 9 of the described symptoms including anhedonia or depressed mood in order to be diagnosed with MDD (Uher et al., 2014). This implies that a total number of 681 possible combinations of symptoms is contemplated for each patient (Akil et al., 2018). Such variety of symptoms and individual differences among patients are clear indications of the heterogeneity of its pathophysiology (Massart et al., 2012).

Hence, currently, the hypothesis of a unitary construct as the cause of MDD has been discarded. Instead, it is believed that a multifactorial etiology of MDD provides a more complete explanation. In fact, several factors have been long associated with the development of MDD including environmental, but also genetic (Nestler et al., 2002). For example, adverse life events, like early-life stress, are considered one of the greatest risk factors (Torres-Berrío et al., 2019; Nelson and Gabard-Durnam 2020). Alternatively, some genetic influences have also been associated with the heritability of MDD, which is estimated to be approximately 38% (Kendler et al., 2006).

Since then, although several theories have been developed, none of them are able to justify the substantial variability of symptoms and risk factors of MDD patients. Therefore, there is a clear need for more integrative theories that recapitulate potentially altered mechanisms leading to such assorted symptomatology. This would be truly beneficial for the development of novel therapies, which could mitigate the enormous burden that MDD entails for our society.

## The Endocannabinoid System

The ECS is a biological modulatory system present in the central nervous system (CNS) of most vertebrates as well as in peripheric tissues. It consists of two main endocannabinoid receptors (ECRs), their endogenous ligands (endocannabinoids) and a number of specialized enzymes for the synthesis and degradation of said ligands. Furthermore, exogenous cannabinoids have also been detailed, which can be synthetic or natural, namely phytocannabinoids. The following section will shortly describe each component of the ECS as well as the most relevant findings in this area.

### Endocannabinoid Receptors

The first identified endogenous component of the ECS was the first cannabinoid receptor (CB1) which was discovered and cloned from the cerebral cortex of rats (Matsuda et al., 1990). In the brain, CB1 receptors are the most common G-protein coupled receptor (GPCR), expressed mainly in neurons and varying greatly across brain areas (Mackie, 2005). Nonetheless, although in much lower concentrations, CB1 receptors are also present in astrocytes, oligodendrocytes and microglia (Stella, 2009; Castillo et al., 2012).

Similarly to most GPCRs, CB1 is essentially located in the cell membrane, particularly in presynaptic axon terminals. There, they are activated by ligands released by post-synaptic neurons upon their depolarization resulting in the inhibition of further neurotransmitter release (Szabo et al., 2000). This retrograde function can resolve into short-term depolarization-induced inhibition of excitatory or inhibitory (DSE and DSI, respectively) transmission (Yoshida et al., 2002; Diana and Marty, 2004), or long-lasting forms of neuroplasticity, like long-term depression (LTD) or potentiation (LTP) (Kellogget al., 2009; Silva-Cruz et al., 2017). Furthermore, CB1 can also act pre-synaptically leading to the activation of the mitogen-activated protein kinase (MAPK) pathway (Bouaboula et al., 1995), suggesting its involvement in cell proliferation and death processes in the hippocampus (Derkinderen et al., 2001). Lastly, CB1 has also been found in alternative subcellular localizations with different functionalities from their plasma membrane equivalents, constituting a subpopulation with distinct pharmacological properties (Robledo-Menendez et al., 2021).

Although the molecular mechanisms of CB1 require further investigation, several studies have linked CB1 function to certain behavioral pathways. Furthermore, CB1 knock-out (KO) mice display an anxiogenic phenotype (Ledent et al., 1999; Zimmer et al., 1999; Martin et al., 2002), suggesting a role for the ECS in anxiety. Interestingly, effects of CB1 agonists are broadly described as biphasic, since their activation in different types of cells can lead to opposing effects on behavior (Busquets-Garcia et al., 2018).

The cannabinoid receptor (CB2) was identified and cloned in 1993 (Munro et al., 1993). It was believed that CB2 was only expressed in immune cells, but their presence in the CNS was demonstrated years later (Van Sickle et al., 2005; Ashton et al., 2006). In light of such findings, an immunoregulatory function of CB2 was proposed. Transgenic models of mice lacking CB2 receptors have contributed greatly to the investigation of its immunomodulatory role. Indeed, CB2 KO mice show exacerbated inflammation (Turcotte et al., 2016). Such findings suggest the fundamental role of CB2 receptors in maintaining immune homeostasis across the organism.

In the CNS, CB2 receptors are present in microglia, macrophages, T and B cells, and natural killers (Matias et al., 2002; Klegeris et al., 2003; Graham et al., 2010; Ramirez et al., 2013). They are a key modulator of neurological activities like nociception, neuroinflammation, and neuroprotection (Malan et al., 2001; Cabral et al., 2008), while, at the same time, its activation is devoid of psychotropic effects. Although the cellular mechanisms of CB2 function are mostly unknown. CB2 are involved in neurological functions such as anxiety, impulsive behaviors, and pain (García-Gutiérrez et al., 2012; Navarrete et al., 2012; Han et al., 2013). Nevertheless, more research is required to clarify the function of CB2 in the CNS.

Even though during the past years numerous studies have investigated the ECS, to date, no further cannabinoid receptors have been reported. Although, some results suggest that certain effects of cannabinoids are not regulated by CB1 or CB2 (Brown, 2007), which has generated some debate regarding the potential existence of a third cannabinoid receptor, nonetheless this remains merely a hypothesis.

### Endocannabinoids

Following the identification of the endogenous cannabinoid receptors CB1 and CB2, research focused on the study of endogenous ligands. This lead to the discovery of the first cannabinoid-like substance N-arachidonoylethanolamide, also known as anandamide (AEA) (Devane et al., 1992). Additionally, a second endocannabinomimetic compound was isolated: 2-arachidonoylglycerol (2-AG). The discovery of these two endogenous cannabinoids (namely, endocannabinoids) reaffirmed the significance of the cannabinoid receptors and their ligands as mediators of a wide variety of biological mechanisms.

Both molecules are lipophilic structures derived from arachidonic acid (Pacher et al., 2006) and they are mainly generated at postsynaptic neurons. Their synthesis is triggered by an increase in postsynaptic intracellular calcium by itself or combined with the activation of postsynaptic GPCRs (Maejima et al., 2001). Upon release, endocannabinoids bind to CB1 and CB2 receptors in the presynaptic membrane with varying affinity. Specifically, AEA seems to be a high affinity, partial agonist of the CB1 receptor, but almost inactive at CB2, whereas 2-AG behaves as a full agonist at both CB1 and CB2 with moderate to low affinity (Sugiura et al., 2000; Pertwee et al., 2010; Di Marzo and De Petrocellis, 2012).

In the brain, the basal levels of 2-AG are approximately 200-fold higher than those of AEA (Sugiura et al., 2006), which indicates its more prominent effects in the CNS. 2-AG is mostly responsible for retrograde signaling via activation of CB1 receptors (Sugiura et al., 1997). It is considered the major mediator of CB1-induced forms of synaptic plasticity such as DSI and long-term hippocampal GABAergic depression (Wilson and Nicoll, 2001; Kim and Alger, 2004). Furthermore, 2-AG is able to activate CB1 receptors present in astrocytes, which eventually results in glutamate release (Navarrete and Araque, 2008) and therefore, mediating neuron-astrocyte communication.

Regarding AEA, it acts as a retrograde messenger and activates CB1 receptors expressed pre-synaptically in glutamatergic terminals (Grueter et al., 2010). This process results in LTD via suppression of glutamate release. Furthermore, AEA participates in “tonic” suppression of GABAergic transmission in the hippocampus (Kim and Alger, 2010). Besides, AEA can act on intracellular CB1 associated with endosomal and lysosomal compartments.

Overall, it is hypothesized that, regarding CB1, AEA represents the “tonic” signaling molecule regulating basal synaptic transmission, whereas 2-AG represents the “phasic” signal which is activated during sustained neuronal depolarization and is responsible for many forms of synaptic plasticity, at least, in the hippocampus (Castillo et al., 2012; Lee et al., 2015). However, these signaling pathways might differ in other brain regions under different physiological or pathophysiological conditions.

When bound to CB2 receptors, endocannabinoids display a series of effects in immune cell function. Interestingly, there is a pronounced contrast between the effects of AEA and 2-AG on immune regulation. 2-AG was found to regulate mechanisms regarding leukocyte recruitment like chemokine release and cell migration (Turcotte et al., 2016). This implies the existence of positive regulation of the immune system by 2-AG. Alternatively, AEA was shown to downregulate leukocyte functions, such as cytokine release and nitric oxide production (Turcotte et al., 2016). Some studies report an increase in production of anti-inflammatory molecules like interleukin (IL) 10 in cells treated with AEA (Correa et al., 2010; 2011). In fact, CB2 receptor agonists like delta-9-tetrahydrocannabinol (THC) and WIN 55,212-2 have only been shown to cause anti-inflammatory effects on leukocytes (Turcotte et al., 2016).

AEA and 2-AG have also been reported to interact with other receptors in the body. AEA acts as an endogenous ligand for transient receptor potential vanilloid 1 (TRPV1) (Starowicz et al., 2007). At pre-synaptic TRPV1s, AEA directly facilitates glutamate release in the striatum (Musella et al., 2009). Alternatively, post-synaptic activation of TRPV1 by AEA also occurs and results in a reduction of biosynthesis of 2-AG (Maccarrone et al., 2008) which, in turn, might lead to LTD (Chávez et al., 2010). In addition, AEA receptor targets include other GPCRs like GPR55 and GPR119 (Sharir et al., 2012). However, these interactions have not yet been fully studied nor understood.

### Metabolic Enzymes

Other key components of the ECS are the catabolic and anabolic enzymes responsible for the synthesis and degradation of endocannabinoids. Briefly, 2-AG is produced by the enzyme diacylglycerol lipase (DAGL) starting from the compound diacylglycerol (DAG) (Murataevaet al., 2014). Alternatively, the biosynthesis of AEA begins from membrane phospholipid precursor N-acyl-phosphatidylethanolamine (NAPE) and it involves the enzymes calcium-dependent or independent N-acyltransferase (NAT or iNAT, respectively) (Jin et al., 2007; Jin et al., 2009) together with NAPE-specific phospholipase D (NAPE-PLD) (Okamoto et al., 2004). However, the exact mechanisms of its synthesis remain under investigation.

Regarding their catabolism, both molecules follow distinct paths. Upon 2-AG reuptake, it is degraded by enzyme monoacylglycerol lipase (MAGL) (Dinh et al., 2002, 2004) which can be found in presynaptic locations and in axon terminals (Gulyas et al., 2004). On the other hand, AEA is degraded by fatty acid amide hydrolase (FAAH) into arachidonic acid and ethanolamine (McKinney and Cravatt 2005) present, particularly, in postsynaptic terminals (Gulyas et al., 2004).

**FIGURE 1:**
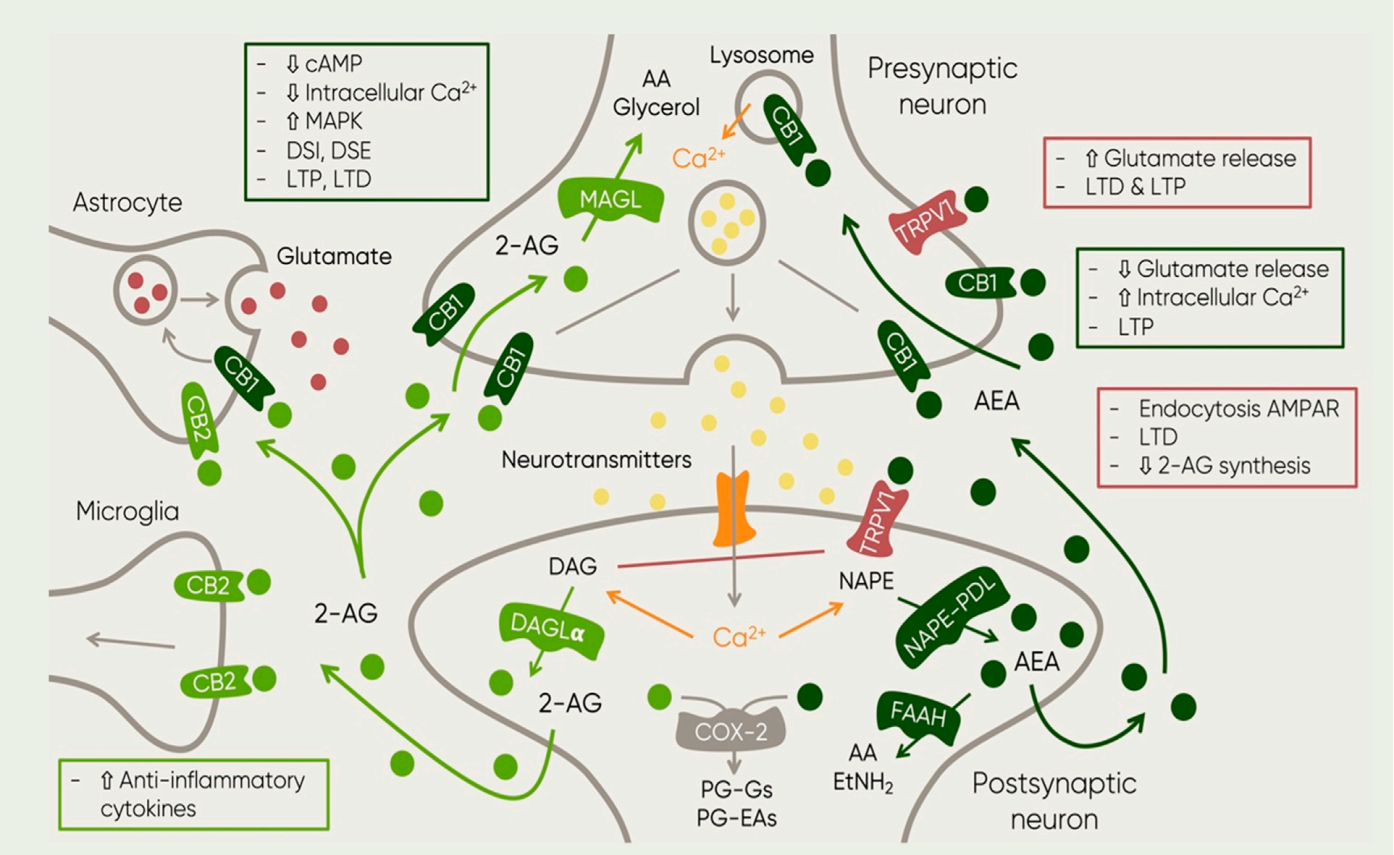
Schematic representation of the main elements of the ECS and their function in the context of neurotransmission. The figure also includes the main synthesis and degradation processes of both endocannabinoids: 2-AG and AEA. The first one is mainly synthesized by the enzyme DAG in post-synaptic neurons as a response to increased excitatory activity (Ludanyi et al., 2011). Upon 2-AG reuptake, it is degraded by enzyme monoacylglycero/lipase (MAGL) (Dinh et al., 2002; Dinh et al., 2004) which can be found in presynaptic locations and in axon terminals (Gulyas et al., 2004). This process results in two major byproducts: AA and glycerol (Dinh et al., 2002). AEA on the other hand, it is synthesized by the enzyme NAPE­ PDL in postsynaptic neurons where it is also degraded by enzyme FAAH (Gulyas et al., 2004). Then, metabolites of AEA and 2-AG undergo further oxidative processes involving cyclooxygenase (COX) and lipoxygenase (LOX) (Vandevoorde and Lambert, 2007). Such process results in the generation of prostag/andins (PGs), in particular, 2-AG and AEA degradation leads to PG-glycerol esters (PG-Gs) and PG-ethanolamides (PG-EAs) (Alhouayek and Muccioli, 2014). The image also shows the receptors at which both endocannabinoids bind. Furthermore, the squares described the resulting mechanisms of the activation of such receptors at different locations. Abbreviations: 2-AG, 2-arachidonoylglycerol; AA, arachidonic acid; AEA, anandamide; cAMP, cyclic adenosine monophosphate; Ca2+, calcium; CB1, cannabinoid receptor 1; CB2, cannabinoid receptor 2; DAG, diacylglycerol; DAGLa; diacylglycerol lipase; DSE, depolarization-induced inhibition of excitatory transmission OSI, depolarization-induced inhibition of inhibitory transmission EtNH21 ethylamide FAAH, fatty acid amide hydro/ase; LTD, long-term depression; LTP, long term potentiation; MAGL, Monoacyclycerol Lipase; MAPK, Mitogen-Activated Protein Kinase; NAPE-POL, NAPE-specific Phospholipase D; NAPE, N-acyl-phosphatidylethanolamine; PG-EAs, prostamides; PG-Gs, prostag/andin-glycero/esters; TRPV1, Transient Receptor Potential Vanilloid.

Importantly, suppression of FAAH and MAGL leads to an activity prolongation of endocannabinoids (Gaetani et al., 2009) resulting in differential effects. On one hand, blocking anandamide degradation reduces pain, inflammation depression and anxiety (Tanaka et al., 2019; Y.; Wang and Zhang, 2017). On the other hand, blockage of 2-AG degradation leads to hypothermia, hypomotility and analgesia (Long et al., 2009). This observation suggested FAAH as a potential pharmacological target and the development of synthetic inhibitors that could potentiate AEA transmission which might prove beneficial for the treatment of disorders like MDD (Fowler, 2015). A schematic representation summarizing the role of the named elements of the ECS in neurotransmission can be observed in Figure 1.

### Phytocannabinoids

Phytocannabinoids are cannabinoids produced in the trichomes of the Cannabis plant (*Cannabis sativa, Cannabis indica and Cannabis ruderalis*). These species contain more than 100 different biologically psychoactive compounds (Aizpurua-Olaizola et al., 2016; Pertwee, 2006). Among them, the most commonly studied due to its strong psychotropic effects is THC. Nonetheless, an increasing number of studies have focused on a different compound named cannabidiol (CBD) since it displays very different pharmacological effects than THC without psychotropic activity. Importantly, later findings unveiled the lack of psychotropic activity of CBD altogether, as well as its absence of reinforcing and addictive properties and a number of characteristics which will advocate for its high potential for therapeutic use (Howlett et al., 2002). Interestingly, the pharmacological effects of CBD go beyond endocannabinoid receptors. For instance, when exerting activity at 5-HTA receptors, CBD induces anxiolytic effects (de Mello Schier et al., 2014; Zanelati et al., 2010). Furthermore, CBD can act as an agonist of vanilloid receptor TRPV1 and therefore influences pain perception, inflammation (Costa et al., 2004). Beyond that, CBD is also known to have affinity for glycine (Ahrens et al., 2009), GABA_A_ (Bakas et al., 2017), adenosine A1 (Gonca and Darıcı, 2015) and nuclear receptors (Scuderi et al., 2014).

In this manner, research then established that THC mainly acts as a partial agonist of CB1 and CB2 (Shahbazi et al., 2020). Alternatively, CBD was found to have reduced binding affinity to both cannabinoid receptors (Pertwee, 2008). However, this compound can also act as a negative allosteric modulator of CB1 and CB2 receptor agonists (Laprairie et al., 2015).

## The Endocannabinoid System and Major Depressive Disorder

As previously mentioned, MDD is a growing global problem. Unfortunately, only a reduced percentage of people with depression achieve a complete remission. As discussed in previous sections, no hypothesis has been able to explain all the signs and symptoms of MDD, since it is a multifactorial disorder involving multiple interlinked mechanisms. Such interconnectivity of pathophysiological mechanisms is believed to manifest as a constellation of symptoms depicting MDD. These include: dysregulation of the HPA-axis, genetic and environmental factors, neurogenesis, and neuroinflammation (Jesulola et al., 2018). Nevertheless, none of these are able to explain MDD’s intricate pathophysiology, nor its symptomatology by themselves. In fact, it is unclear whether some observed dysregulations are a direct cause or rather a consequence of this disorder. This last challenge obstructs enormously the development of very necessary novel medications or treatments for MDD.

Fortunately, the growing research in the field of the ECS has been opened a promising perspective. As stated in previous sections, the proper interplay between all the elements of the ECS is essential for the homeostatic maintenance of a number of physiological, cognitive, behavioral, and emotional processes (Mechoulam and Parker, 2013). Therefore, when dysregulation of the ECS occurs, cognitive deficits might arise. Particularly, animal research has established a clear symptomatic overlap between ECS alterations and MDD (Hill and Gorzalka, 2005). Importantly, this phenomenon has also been observed in humans. For example, female patients diagnosed with depression presented altered endocannabinoid levels in serum compared to healthy participants (Hill et al., 2008a). Accordingly, the participation of the ECS in the pharmacology of antidepressant drugs has also been reported (Hill et al., 2008b). This evidence indicates a clear role of the ECS in the pathophysiology of MDD in humans. Besides, these ECS alterations seem to occur in a brain/region-dependent manner in depressed individuals, which would explain the variety of symptoms contemplated at the time of diagnosing MDD.

Particularly, the contribution of CB1 receptors to MDD has received broad attention. Animal experiments have established the important role of CB1 as a mood regulator. This can be observed in CB1-KO rodents, which show depressive-like phenotypes (Valverde and Torrens 2012). For example, ablation of CB1 results in anhedonia (Sanchis-Segura et al., 2004), passive stress-coping behavior (Steiner et al., 2008), and higher sensitivity to develop depressive-like symptoms (Haller et al., 2002; Martin et al., 2002).

Furthermore, it is important to consider the evidence regarding the impact of cannabinoid agonist and antagonists administration on MDD. For instance, enhancement of CB1 signaling via administration of HU-210, a potent CB1 agonist, results in antidepressant effects (Hill and Gorzalka, 2005). Additionally, methanandamine, a stable AEA derivative, when applied through stereotaxic injections into the prefrontal cortex (PFC) of rats, was able to induce anxiolytic effects mediated by CB1 (Rubino et al., 2008). Lastly, synthetic agonist WIN55,212-2 was found to induce these effects via modulation of the 5-HT neuronal activity (Bambico et al., 2007). Nevertheless, these results appear to be biphasic, since the application of high doses of CB1 agonists can result in increased anxiety-like behavior. In fact, in humans, functional magnetic resonance imaging (fMRI) revealed that THC can reduce amygdalar reactivity of healthy volunteers exposed to threat signals (Phan et al., 2008), an effect that is commonly observed after administration of anxiolytic drugs like benzodiazepines. In contrast, intravenous administration induced psychotic-like symptoms and anxiety (D’Souza et al., 2004). Therefore, it is hypothesized that oral administration might contribute to lower peak serum concentrations, potentially helping to avoid aversive emotions resulting from high levels of CB1 activation (Moreira et al., 2009).

Accordingly, there is evidence that CB1 antagonism can increase aversive responses in animal models anxiety and depression. The best example is rimonabant, a CB1 antagonist initially marketed as a treatment for depression that resulted in severe adverse psychiatric events in the patients (Mitchell and Morris 2007). Posterior research revealed that, indeed, rimonabant was an anxiogenic substance that not only induced a depressive-like phenotype in laboratory animals, but it also lead to a series of molecular alterations such as decreased serotonin levels in the frontal cortex, reductions in hippocampal cell proliferation and survival, and increased concentrations of pro-inflammatory cytokines (Beyer et al., 2010). Furthermore, AM-251 a different CB1 antagonist, has also been observed to possess anxiogenic properties (Rodgers et al., 2005). In addition, administration of both antagonists; AM-251 and AM-630, significantly reverses the antidepressant effects of WIN55, 212-2 in animals exposed to social-isolation-induced stress (Haj-Mirzaian et al., 2017). Unfortunately, the mechanisms underlying the effects of both CB1 receptor agonists and antagonists remain unknown and must be explored in more detail at the mechanistic level. The supplementary materials include a table summarising the preclinical and clinical studies confirming association between the endocannabinoid system and MDD (see Supplementary Table S1).

Nevertheless, a considerable number of studies has researched the implications of endocannabinoid components in several mechanisms involved in MDD without directly studying their impact on this disorder per se. Hence, this existing literature might be helpful at the time of understanding the role of the ECS in proposed etiologies of MDD, particularly, genetic factors, dysregulation of the HPA-axis, neurogenesis, and neuroinflammation (Jesulola et al., 2018). In the following sections, the present review will focus on the role of the ECS for each theory regarding the pathophysiology of MDD.

### Genetic Factors

Genetic studies are a great source of evidence confirming the role of the ECS in MDD. Together with other genetic factors external to the ECS, epidemiological work of past decades has provided evidence of a certain degree of MDD heritability (Mondimore et al., 2006). Hence, it was hypothesized that variations in certain genes could be substantially responsible for the development of MDD. Nonetheless, up to date, no single genetic mutation is necessary nor sufficient to explain MDD, instead, each gene variation contributes only a reduced fraction of the total risk (Major Depressive Disorder Working Group of the Psychiatric GWAS Consortium et al., 2013).

In humans, genetic studies have investigated single nucleotide polymorphisms in the CB1 gene (*CNR1*) associated to depressive phenotypes and responses to antidepressants. For example, presence of the G allele of rs806371 *CNR1* gene polymorphism is higher in individuals with MDD (Mitjans et al., 2013). On the same line, the G allele of the CNR1rs1049353 polymorphism has been associated to antidepressant resistance (Domschke et al., 2008). Alternatively, the minor C allele of rs2023239 displayed a protective influence against MDD (Icick et al., 2015).

Nevertheless, studies associating gene variants and MDD often encounter inconsistent data. A recent report analyzed several candidate genes previously implicated in high prevalence of MDD, among them, a *CNR1* variant (Gonda et al., 2018). It was observed that most polymorphisms, including those in *CNR1,* showed increasing relevance for MDD in participants with higher exposure to recent negative life events. Possibly, the lack of accountability for environmental factors like stress could bias their contribution to MDD and cause the results to become irreproducible. As a matter of fact, a recent meta-analysis by Kong et al. (2019) revealed no association between CNR1rs1049353 or CNR1 triple repeat with increased risk of MDD when combining all available literature.

In contrast, a significant correlation was found between all four genetic models of the CB2 gene (*CNR2*) polymorphism CNR2rs2501432 and MDD. This was first observed by Onaivi and collaborators (2008), who reported a significant association between such *CNR2* polymorphism and depressed patients. Furthermore, a recent article has linked *CNR2* R63Q variation to a greater sensitivity towards childhood trauma and overactivation of the HPA-axis (Lazary et al., 2019). Interestingly, the article also describes the involvement of a FAAH gene polymorphism in the susceptibility to trauma observed in Lazary et al. (2016).

In conclusion, the role of *CNR1* in MDD should not be dismissed and further research with wider coverage is needed to evaluate its impact. However, this finding positions *CNR2* as a key factor in the development of MDD. Hence, pharmacological modulation of *CNR2* ligands might be a potential therapeutic approach for this disorder. Importantly, the association of endocannabinoid receptors to MDD is not sufficient to explain the entirety of its pathophysiology. Therefore, such results should be interpreted with caution, while contemplating the essential role of environmental factors in its etiology.

### Stress and the HPA-Axis

Stressful life events are considered the main predisposing factor for the development of psychiatry disorders like anxiety and MDD. In mammals, the stress response is mediated by the HPA-axis, resulting in a cascade of events involving a series of hormones; like corticotropin-releasing hormone (CRH) and adrenocorticotropic hormone (ACTH); ultimately resulting in the release of glucocorticoids into the bloodstream (Smith and Vale, 2006). Termination of the stress response is achieved by resetting the HPA-axis via negative feedback mechanisms. On this basis, hyperactivity of the HPA-axis has been suggested as a mechanism leading to stress vulnerability shown by MDD patients. Certainly, these patients show inadequate HPA-axis suppression when exposed to stress or exogenous glucocorticoid administration compared to healthy controls (Gillespie and Nemeroff, 2005; Juruena, 2014). Interestingly, this dysregulation of the HPA-axis seems to be related to endocannabinoid signaling (Hillard et al., 2016).

For example, CB1 receptors are believed to play a major role in HPA-axis regulation. This hypothesis arose from the observation of high basal corticosterone levels and HPA hyperactivity in CB1 KO animal models (Barna et al., 2004). This indicated a clear role for CB1 in the inhibition of the HPA-axis and therefore, in the termination of the stress response. On the other hand, HPA-axis dysregulation can also influence the ECS via alterations in endocannabinoid synthesis and CB1 expression, both involved in stress-related symptoms (Micale and Drago, 2018). This suggests the existence of a bidirectional link between the two systems.

Furthermore, the role of the ECS was investigated in existing stress models associated to anxiety-like behavior in rodents. It was observed that chronic stress induced a reduction of AEA levels in the amygdala and hippocampus (Patel et al., 2005; Wang et al., 2012) via increased activity of FAAH (Navarria et al., 2014; Gray et al., 2015), which is consistent with previous evidence supporting the anxiolytic properties of AEA (Patel and Hillard, 2006). This reduction was attributed to the elevated serum corticosterone content (Hill et al., 2009) through a CRHR1-mediated mechanism (Gray et al., 2016) resulting in the generation of anxiety (Gray et al., 2015). Nevertheless, the obtained results depended largely on the type of stressor. Furthermore, exposure to repeated stressors increased 2-AG content throughout regions in the CNS (Micale and Drago 2018) probably mediated by decreased expression of MAGL (Sumislawski et al., 2011). This is in accordance with the observed decrease in AEA levels following stress exposure, since its interaction with TRPV1 results in downregulation of 2-AG synthesis (Maccarrone et al., 2008). However, this link has not been empirically demonstrated.

Importantly, following chronic unpredictable stress, CB1 undergoes widespread downregulation and desensitization in limbic areas like the hippocampus, hypothalamus, amygdala and nucleus accumbens (Wamsteeker et al., 2010; Wang et al., 2010; Lee and Hill, 2013). In contrast, many studies have found an upregulation of CB1 in PFC (Zoppi et al., 2011; Lee and Hill, 2013). Unfortunately, the exact mechanism by which this CB1 regulation occurs remains under speculation. However, it is consistent with the observation that CB1 activation decreases GABAergic transmission in said limbic areas, contributing to the termination of the stress response (Martin et al., 2002; Hill et al., 2011). Particularly, CB1 blockade leads to increased plasma corticosterone (Wade et al., 2006). Furthermore, the activation of CB1 on noradrenergic signaling represents an important mechanism for stress adaptation (Wyrofsky et al., 2019). Chronic HPA-axis-induced stimulation of noradrenergic areas such as the locus coeruleus increases anxiety and depressive-like behavior (Valentino and Van Bockstaele, 2008). Importantly, hyperactivity of the ECS via CB1 signaling due to chronic stress might lead to increased noradrenergic activity in the PFC and thus, contributing to such behaviors (Wyrofsky et al., 2019).

On this basis, CB1 dysregulation within specific brain areas would lead to a hyperactive HPA-axis, which is precisely what is observed in CB1 KO animals (Daniela Cota et al., 2007; Steiner et al., 2008). CB1 KO animals express higher anxiogenic-like behavior and increased stress-induced ACTH levels, as well as anxiolytic drug resistance (Cota, 2008). Accordingly, hyperactivity of the HPA-axis is one of the most consistent biological evidence in MDD in both clinical and pre-clinical studies (Sánchez et al., 2001; Heim and Nemeroff 2002). This suggests a clear role for CB1 as a direct contributor to the termination of the HPA-mediated stress response and therefore, as a key partaker in the pathophysiology of depressive symptoms.

With regards to CB2 receptors, despite their expression in areas involved in the stress response, few studies have explored their implications in HPA-axis signaling. In these, results are contradictory. Some stress-induced depressive-like behavior models have described no changes in CB2 levels, whereas others encountered a decreased CB2 hippocampal expression (Marco et al., 2017). However, unlike CB1, manipulation of CB2 receptors does not alter plasma concentrations of corticosterone after exposure to stress (Zoppi et al., 2014). Such findings speak against a direct relationship between the HPA-axis and CB2 receptors, although it is possible that CB2 might regulate the activity of the HPA-axis through other indirect mechanisms. Indeed, a recent article has linked an existing polymorphism of the gene encoding CB2 to greater sensitivity for childhood trauma and overactivation of the HPA-axis (Lazary et al., 2019). This process seems to be mediated through neuroinflammatory mechanisms influenced by CB2. This observation is in accordance with previous findings showing that CB2 KO animals display exacerbated stress-influenced neuroinflammatory responses (Zoppi et al., 2014).

**FIGURE 2:**
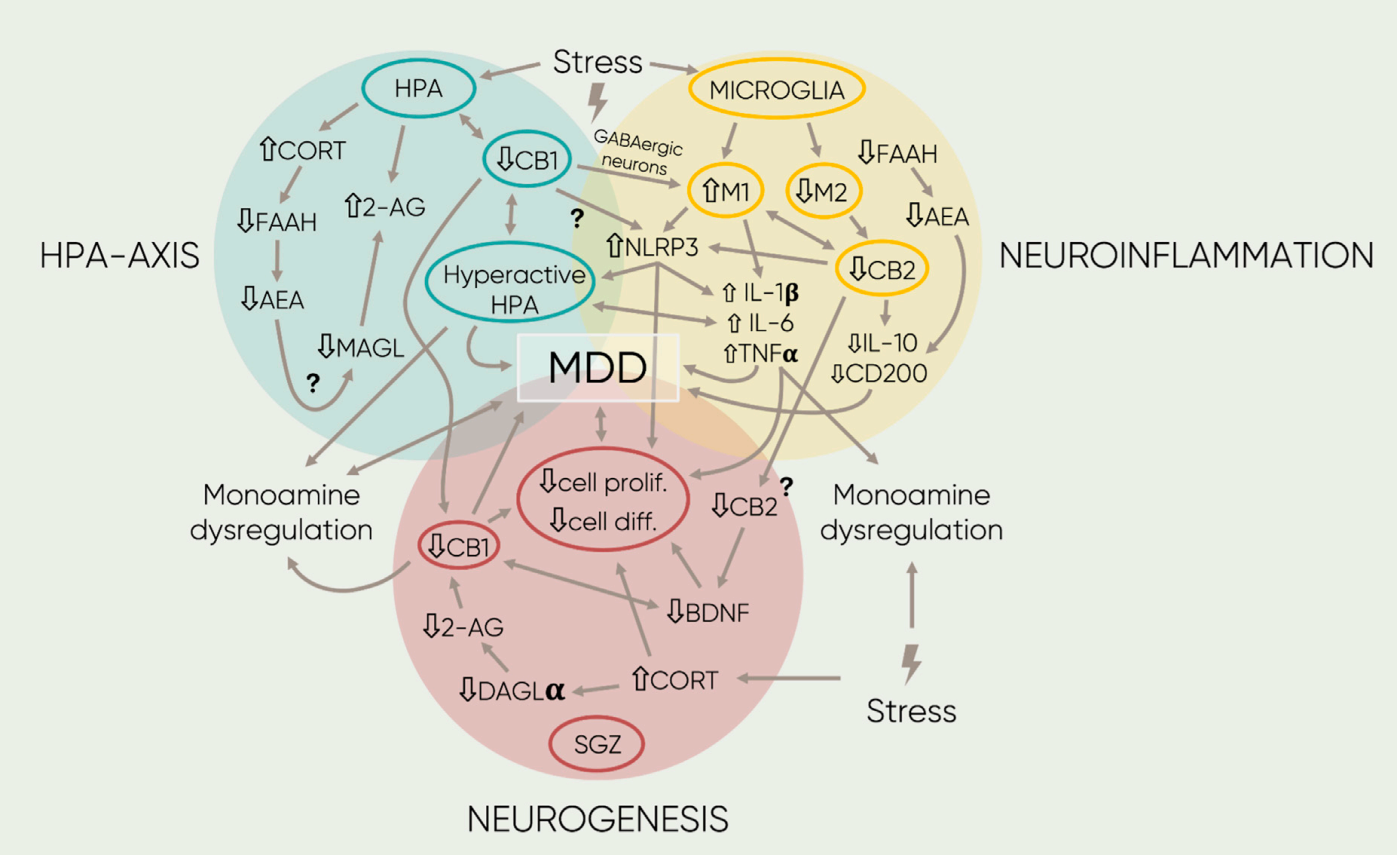
Schematic representation of the main findings regarding the association of the ECS and MOD. The four different proposed etiologies of MOD are represented by the three colored circles. Overlaps between circles symbolize interactions between mechanisms. Inside, each circumference highlights the central components in each field. The single-arrowed lines represent direct observed influences between factors whereas the double-arrowed ones, bidirectional links. Next to the text, arrows represent down- or upregulation of the component or signaling mediated by receptors. Furthermore, the lightning symbols stand for different forms of stress (early-life, acute, or chronic stress; see text for more details). Lastly, the question marks beside the arrowed lines indicate potential links that remain to be researched. 2-AG, 2- arachidonoylglycerol; AA, arachidonic acid; AEA, anandamide; BDN F, brain derived neurotropic factor; CB1, cannabinoid receptor 1; CB2, cannabinoid receptor 2; CORT, cortisol/corticosterone, DAGLa, diacylglycerol lipase; diff, differentiation; FAAH, fatty acid amide hydrolase; IL-1,6′, Interleukin 1/l; IL-6, interleukin 6 MAGL, Monoacyclycerol Lipase; Ml, microglia phenotype 1; M2, microglia phenotype 2; N LRP3, family pyrin domain containing 3; pro/if, proliferation; TNFa, tumor necrosis factor a.

All together, these findings suggest the involvement of the ECS; particularly, the CB1 receptor and endocannabinoids AEA and 2-AG, as a fundamental regulatory system involved in the termination of the stress response by mediating negative feedbacks controlling HPA-axis’ activity. Therefore, these ECS components have a direct role in anxiety-like behavior and vulnerability to stress, and they must be regarded as important contributors to the pathophysiology of MDD. All the above-mentioned mechanisms are graphically described within the blue area of Figure 2.

### Neuroinflammation

Among the other potential altered circuitries in MDD, neuroinflammation has received increasing attention (Troubat et al., 2020). Since the first observations, several studies have described a strong relationship between depressive symptomology and altered presence of pro-inflammatory markers. Hence, research has focused on the involvement of the immune system in the CNS, which consists of glial cells.

As the resident macrophagic cells and main form of innate immune defense in the CNS, microglia were expected to play a crucial role in this relationship. Microglia are responsible for the release of chemokines and cytokines as a response to physical insults or infectious agents. Upon activation, microglia can evolve into two possible phenotypes, known as M1 and M2, which might include specific features under different pathological insults (Ma et al., 2017). The first, M1, is in charge of the initiation of the inflammatory response through the release of pro-inflammatory mediators like IL-1β, IL-6, IL-8 and tumor necrosis factor α (TNFα) (Lively and Schlichter, 2018). Furthermore, they control the recruitment of additional central and peripheral immune cells to the site of infection or brain damage. Alternatively, the second phenotype, M2, is involved in the prevention of M1-induced neuronal damage and toxicity through secretion of anti-inflammatory cytokines like IL-4, IL-10 and transforming growth factor (TGF) (Koscsó et al., 2013). This M1/M2 phenotype theory has been found to be an oversimplification of this cytological process based on experimental evidence (Ransohoff 2016). However, this terminology is useful when characterizing microglia states (pro-inflammatory and neuroprotective) and will continue to be used for the purpose of this review.

Due to their primary role in neuromodulation of the inflammatory response, microglia have become the center of attention as a key component in the inflammatory etiology of MDD (Yirmiya et al., 2015). Evidence supporting these theories is provided by clinical and preclinical research. For instance, elevated levels of proinflammatory cytokines released by microglia are correlated to the severity of depressive symptomatology in MDD patients (Haapakoski et al., 2015). Furthermore, rodents exposed to chronic stress paradigms display microglia hyper-ramification and overactivation in several brain regions (Sugama et al., 2007; Tynan et al., 2010; Hinwood et al., 2013).

Particularly, IL-6 and C-reactive protein (CRP) are the most strongly associated to anhedonia (Felger et al., 2018). In accordance, administration of antidepressants directly impacts microglia signaling and IL-6 production (Hashioka et al., 2007). A more recent study has also linked the imbalance of microglial pro- and anti-inflammatory conditions through a mechanism involving brain-derived growth factor (BDNF), more specifically, via a BDNF-TrkB-dependent pathway in the hippocampus (Liu et al., 2019). Interestingly, the authors found a sex-specific relationship between BDNF and microglial inflammatory biomarkers, a mechanism that could become very important for sex differences in depression. On this basis, some researchers have suggested that MDD could start to be considered a microglial disease (Yirmiya et al., 2015).

The presence of CB2 receptors in microglia switched the attention to the potential role of the ECS in glia-mediated neuroinflammation involved in MDD. CB2 has been described to possess general anti-inflammatory properties. Genetically modified rodents with overexpression of CB2 show reduced stress-induced pro-inflammatory cytokine TNFα and enzyme COX-2 (Zoppi et al., 2014). In parallel, CB2 KO animals display exacerbated neuroinflammatory responses to chronic stress (Zoppi et al., 2014).

CB2 receptors are expressed in microglia in a state-dependent manner (Carlisle et al., 2002). Specifically, they are not present in resting microglia but rather in fully active cells and intermediary stages. In the past decade, its presence has been associated to induction of M2 polarization (Tanaka et al., 2020). For example, administration of synthetic CB2 agonists alleviate neuroinflammation by enhancing the conversion from M1 microglia into the M2 phenotype (Tao et al., 2016; Luo et al., 2018). Importantly, a downregulation of inflammatory cytokines and upregulation of anti-inflammatory mediators were paired to M2 polarization (Tao et al., 2016). These mechanisms were not observed when animals were co-treated with CB2 synthetic antagonists. Such results evidence the seemingly important role of CB2 receptors in microglia-induced neuroprotection in the presence of inflammation.

Furthermore, it appears these anti-inflammatory effects of CB2 might be initiated by AEA. Administration of AEA reduces neuron toxicity by downregulating IL-1B and IL-6 in *in vitro* activated microglia via CB2 receptors (Malek et al., 2015). At the same time, it exerts neuroprotective properties via production of IL-10 (Correa et al., 2010), and upregulation of CD200 receptor, known to suppress microglial inflammatory response (Manich et al., 2019). Moreover, pharmacological inhibition of FAAH also leads to anti-inflammatory effects *in vitro* via increased levels of AEA (Tanaka et al., 2019).

Although these observations have been widely described in pharmacologically induced neuroinflammation, the immunological role of the ECS in the context of MDD models is understudied. However, two recent studies have investigated this relationship. First, Chen et al. (2018) reported that FAAH inhibition resulted in alleviation of pro-inflammatory response to acute stress. This is in accordance with previous studies reporting anti-inflammatory properties of AEA. Second, Lisboa et al. (2018) observed that administration of selective cannabinoid agonist WIN55,212-2 was able to reverse social stress-induced neuroinflammation and anxiety-like symptomatology. Although, since named agonist could act on both cannabinoid receptors, whether this action occurred through mediation of CB1 or CB2 is unclear.

Regarding CB1, despite its low expression in glial cells, some studies have shown that this receptor is also able to regulate immune function through distinct mechanisms. For instance, specific CB1 KO of forebrain GABAergic neurons *in vivo* lead to pro-inflammatory microglia phenotypes without significant cognitive deficits (Ativie et al., 2018). This finding provides evidence of the role of CB1 in neuron-glia communication with a crucial involvement of GABAergic neurons. Furthermore, a recent research work observed that chronic stress increased microglia activation in CB1 KO animals, which correlated with the severity of depressive-like symptoms (Beins, 2020). Hence, it has been hypothesized that CB1 might be an indirect mediator of microglia-induced inflammation. Although, how CB1 signaling can regulate microglial activity remains uncertain and requires further research.

For this, it is important to consider the potential interplay between HPA-axis and microglial neuroinflammation. As mentioned earlier, HPA-axis hyperactivity is observed across animal models of MDD, as well as in human patients. Increased glucocorticoid release has been found to induce an overproduction of pro-inflammatory cytokines through microglia activation (Nair and Bonneau, 2006; Sorrells and Sapolsky, 2007). In the same manner, an immune modulation of the HPA-axis has also been suggested following immunology research of viral infections (Silverman et al., 2005). However, the exact mechanisms underlying chronic stress and microglia activation remain under discussion. A recent article described a potential link between HPA signaling and neuroinflammation. Feng et al. (2019) observed that elevated corticosterone levels after chronic stress increased the release of pro-inflammatory elements like IL-1β and IL-18 in activated hippocampal microglia. Particularly, they observed that this process was mediated by nuclear factor kappa B (NF-κB) and the Nod-like receptor protein (NLRP3) namely, NF-κB-NLRP3 pathway. This study provides a new and valuable insight into the relationship between stress, the HPA-axis and microglia-mediated neuroinflammation.

Although much less investigated, the ECS might also be related to this mechanism. Recent research has found that administration of two phytocannabinoids; CBD and cannabigerol produced anti-inflammatory effects by reducing NF-κB activation among other mechanisms (Mammana et al., 2019). Furthermore, previous work had stablished a neuroprotective role for CB2 via inhibition of the NLRP3 inflammasome in autoimmune encephalomyelitis models (Shao et al., 2014). This process might be mediated via the CB2-induced inhibition of NF-κB activation in microglia (Zoppi et al., 2014). Additionally, CB1-mediated effects of 2-AG have been linked to neuroprotective functions via inhibition of NF-κB (Panikashvili et al., 2005). Likewise, AEA has been described to enhance production of IL-10 in activated microglia and inhibiting NF-κB activation. More recently, in the context of liver inflammatory disease, CB1 receptors have been reported to mediate macrophage NLRP3 expression and inflammation (Yang et al., 2020). This evidence suggests a potential role for the ECS as a bridge in the crosstalk between the HPA-axis and neuroinflammation *via* the NF-κB-NLRP3 pathway. However, effects of the ECS in modulation of microglia-mediated neuroinflammation as a result of chronic stress, particularly regarding the NLRP3, remains to be studied and requires much further research.

Importantly, a novel field of research has emerged in recent years, focusing on polyunsaturated fatty acids (PUFAs) and their potential as a treatment for MDD. PUFAs are lipid derivatives of omega-3 or omega-6 that act as precursors for endocannabinoids. Particularly, supplementation with two main PUFAs, namely, eicosapentaenoic acid (EPA) and docosahexaenoic acid (DHA) has been extensively investigated (Mozaffari-Khosravi et al., 2013; Marangell et al., 2003). Indeed a recent meta-analysis confirmed the beneficial effects of omega-3 PUFAs on depression symptoms (Liao et al., 2019). Co-administration of DHA and EPA or EPA alone are able to reduce inflammation through several mechanisms. Firstly, they both can reduce the production of proinflammatory cytokines like TNFα, IL-1β, IL 2 and IL-6 (Caughey et al., 1996). Secondly, they have been described to suppress NF-κB signaling (Horowitz et al., 2015). Lastly, administration of a variety of omega-3 PUFAs reduced inflammation *via* activation of macrophage autophagy and attenuation of NLRP3 inflammasome (Shen et al., 2017). These findings strongly confirm the endocannabinoid regulation of neuroinflammatory processes, and they provide an opening to a new research field in the context of psychiatric disease like MDD.

Overall, the abovementioned evidence supports the theory that hypofunction of the ECS directly impacts neuro-immune modulatory pathways in the CNS, leading to pro-inflammatory processes mediated by microglia. Specifically, CB2 seems to be directly involved in promotion of anti-inflammatory mechanisms mediated by AEA signaling. Nevertheless, the involvement of CB2 in stress-induced inflammation remains unknown. Alternatively, CB1 has been suggested to indirectly modulate neuro-immune responses by mediating neuron-glia communication in the CNS. Such interactions between the immune system, the ECS and MDD symptomatology are depicted in the yellow area in Figure 2.

### Neurogenesis

Adult neurogenesis is a neurobiological process by which neurons are continually generated within the CNS throughout an organism’s life. Specifically, the subgranular zone (SGZ) of the dentate gyrus of the hippocampus has been established as the primary area involved in this process (Bond et al., 2015). Notably, the exact biological role of neurogenesis in cognition and behavior remains unknown.

Since the discovery of hippocampal atrophy in untreated MDD patients (Sheline et al., 1996), it was hypothesized that loss of neurogenesis in the dentate gyrus could significantly impact the development of this disorder. In fact, successful treatment with antidepressants and most therapies is shown to induce hippocampal neurogenesis (Praag et al., 2000; Malberg and Duman 2003; van). This phenomenon seems to be mediated by BDNF, since it is found to be reduced in animals models of MDD (Autry et al., 2009), while simultaneously recovered by antidepressant administration (Santarelli et al., 2003). However, neurogenesis ablation in experimental animals does not always induce depressive symptoms (Jayatissa et al., 2010). On this line, some antidepressants show neurogenesis-independent mechanisms (David et al., 2009). Due to such conflictive results, it is currently hypothesized that neurogenesis might be a key restorative mechanism for hippocampal structure and function which might indirectly result in alleviation of MDD symptomatology (Hanson et al., 2011). Therefore, when disrupted, it could theoretically participate in the etiology of MDD even though it is unlikely to cause the entire mood disorder. In the present, however, neurogenesis is still considered a significant contributing factor to the pathophysiology of MDD (Jesulola et al., 2018).

During the past decades, research has confirmed the involvement of the ECS in hippocampal proliferation, starting with the extensive expression of endocannabinoid entities in neuronal progenitor cells (Aguado et al., 2006). Specifically, CB1 has been established as a direct mediator of adult neurogenesis. For example, administration of high selective agonists of CB1 promotes neural proliferation in the SGZ (Andres-Mach et al., 2015). Accordingly, this effect was prevented by CB1 synthetic antagonist AM251 and not present in CB1 and CB2 double KO animals (Hutch and Hegg 2016). Furthermore, DAGLα KO rodents exhibited decreased 2-AG levels, as well as impaired neurogenesis in the dentate gyrus (Gao et al., 2010). Therefore, CB1 activation via 2-AG agonism seems to play a major role in this biological process. However, the exact mechanism by which CB1 might induce neurogenesis is under discussion, although some pathways have been described (Zimmermann et al., 2018).

Relevant to depressive-like behavior, CB1 KO mice were observed to be more vulnerable to stress-induced depressive-like responses with a high susceptibility for anhedonia (Martin et al., 2002). This symptomatology was later associated to downregulation of BDNF expression in the hippocampus (Aso et al., 2008). Such findings suggested a clear involvement of both, CB1 and neurogenesis in the development of depressive disorders.

Conversely, administration of CBD prevented detrimental effects of chronic stress and increased hippocampal proliferation via CB1 activation (Wolf et al., 2010; Campos et al., 2013). Another recent study reported that CBD administration could reverse the effects of chronic stress, facilitating neurogenesis and dendritic remodeling (Fogaça et al., 2018). However, whether anxiolytic effects of CBD are directly a consequence of improved neurogenesis or other unrelated endocannabinoid mechanisms cannot be concluded. These observations also occurred when considering endocannabinoids. For example, prevention of 2-AG degradation via blockade of MAGL results in an enhancement of neurogenesis and heightened antidepressant-like effects in mice exposed to chronic stress (Zhang et al., 2015). In addition, DAGLα KO animals displayed reduced endocannabinoid levels in the hippocampus, as well as impaired neurogenesis and anxiety-like behavior (Jenniches et al., 2016). Nonetheless, the cause-consequence relationship between the observed depressive symptoms and loss of neurogenesis cannot be established from this study. In summary, CB1 is hypothesized to contribute to anti-depressive effects paired with neurogenic mechanisms in the hippocampus.

A more complex role of CB2 in adult neurogenesis has been suggested. Unlike CB1 KO, CB2 KO animals display a stable adult neurogenesis (Mensching et al., 2019). Although, these findings are contradictory to previously reported observations indeed showing altered neurogenesis in this strain (Palazuelos et al., 2006). This perhaps could be due to the age difference among rodents used for the studies. Regardless of this contradiction, CB2 agonism has been related to indirect potentiation of neurogenesis through its role as a homeostasis regulator, normalizing processes like apoptosis, oxidative stress, and neuroinflammation (Avraham et al., 2014; Shi et al., 2017). For instance, in an Alzheimer’s disease model, administration of CB2 agonist MDA7 induced neurogenesis together with improved hippocampal synaptic plasticity and regulated microglial activation (Wu et al., 2017). Interestingly, microglia are able to regulate this process via the release of cytokines and chemokines that may act as stimulants or suppressors of neurogenesis (Sato 2015). In this manner, pro-inflammatory mediators like IL-1β can inhibit processes such as cell proliferation and differentiation in the dentate gyrus (Wu et al., 2013). In particular, chronic stress in rodents produces alterations in microglia activation, which contributes to the loss of neurogenesis (Kreisel et al., 2014). Alternatively, microglia can also release BDNF, thereby supporting survival of novel neurons (Ferrini and De Koninck 2013). Whether this mechanism is directly regulated by CB2 remains unclear. Nevertheless, more recently it has been observed that activation of CB2 receptors by selective agonist JWH133 upregulated microglia expression of BDNF in the lateral ventricular tissue (Tang et al., 2017). Though this finding has shed some light on the topic, further research is still required before drawing solid conclusions.

Neurogenesis is also related to HPA function in pathological conditions. For example, animals with suppressed neurogenesis display a hyperactive HPA-axis associated to depressive-like behaviors (Schloesser et al., 2009). Given the important role of the hippocampus in termination of HPA-mediated stress response, it was hypothesized that disturbances in neurogenesis could negatively alter the negative feedback loop regulating HPA activity. Simultaneously, chronically elevated glucocorticoid concentrations as a result of a hyperactive HPA also leads neurogenesis disruptions (Anacker et al., 2013).

Interestingly, changes induced by stress or inflammation may affect neurogenesis *via* the NF-κB pathway (Anisman et al., 2008), which is, at the same time, the suggested mechanism bridging HPA activity and neuroinflammatory processes. Furthermore, pro-inflammatory cytokines can additionally stimulate the HPA-axis to release glucocorticoids, which, in turn, suppress neurogenesis (Liu et al., 2003). Hence, it can be concluded that there is a strong interconnection between the ECS and altered mechanisms observed in MDD.

Overall, these results indicate a role for the ECS in modulation of hippocampal cell proliferation and cell differentiation. On one hand, CB1 and 2-AG signaling seem to be essential components in the stimulation of neurogenesis. On the other, CB2 might participate as a key regulator under pathological conditions by exerting neuroprotective mechanisms towards neurons via immune system regulation. These mechanisms are depicted within the red area in Figure 2. In conclusion, the presented data suggest a clear involvement of the ECS in the neuroprotective effects of hippocampal neurogenesis during the development of MDD.

## Discussion and Future Perspectives

The present work aimed to be an integrative review about the role of the ECS in the pathophysiology of MDD. As previously described, all the currently hypothesized etiologies for MDD, whether they are genetic, neuroendocrine, immunological, or cytogenetic, seem to rely on the correct functioning of endocannabinoid signaling. In fact, a number of interactions between studied factors can already be explained through the involvement of the ECS. Figure 2 aims to summarize all the discussed findings in a cohesive mind map. As it can be observed, the different proposed etiologies of MDD—HPA-axis, genetic factors, neuroinflammation, and neurogenesis—can all be integrated into the context of endocannabinoid signaling. In addition, the figure illustrates the potential links that remain to be explored. Among them, the possible role of the NF-κB-NLRP3 should be highlighted as an important factor in the mediation of the multiple described systems. Moreover, given the intricacy of the mechanisms underlying stress-related conditions, further studies are essential to evaluate the role of different players, such as TRPV1 receptors and other GPCRs; diverse neuronal subpopulations, i.e., GABAergic vs glutamatergic; and even considering brain regions, which could interact with each other to regulate mood and cognitive aspects involved in MDD.

This image is a clear example of the complex nature of MDD as well as indication of the current need for interdisciplinary work. Furthermore, it has been described that a sole etiology or a unitary construct as the cause of MDD cannot explain the constellation of symptoms exhibited by depressed patients. Hence, the unification of diverse research fields must occur in order to advance in our comprehension of MDD and other complex etiologies like schizophrenia, post-traumatic stress disorder, or autism spectrum disorder.

As a matter of fact, the complexity of the ECS might be useful when searching for connections between pathological pathways. Similarly to MDD, its function cannot be presented as a list of individual items, but it should be regarded as a wide interconnected network. One that is sensitive to environmental factors, such as stress, which might threaten the integrity of brain homeostasis. It is evident that the correct functioning of the ECS is imperative for maintaining mental health. However, despite being an ever-growing acclaimed research field, further preclinical and clinical studies are crucial for a better understanding of its mechanisms. For instance, the role of GPR55 as an expanded endocannabinoid receptor remains controversial. Moreover, GPR18 has not been investigated, although its presence in microglia suggests a potential role as a neuro-immune regulator. A recent review discusses in depth the so called “expanded” ECS or endocannabinoidome, including a number of elements overlapping with pathways attributed to the ECS (Cristino et al., 2020). The work of Cristino et al. (2020) certainly illustrates the complexity of cannabinoid mechanisms and further highlights the importance of interdisciplinary work at the time of researching this phenomenon.

Such complexity deeply challenges the development of cannabinoid-based therapies. Their characteristic chemical promiscuity can give rise to unexpected side effects. For instance, based on *in vitro* and *in vivo* studies, it was believed that synthetic inhibitors of FAAH could be a potential treatment for depressive and anxiety disorders (Gunduz-Cinar et al., 2013). Therefore, a variety of them were synthesized and tested clinically. Nonetheless, a vast number of them were quickly suspended due to their devastating side effects. Particularly, a famous case reported severe adverse effects in 5 patients and at least one death during a drug trial in France (Kaur et al., 2016). Furthermore, rimonabant, a selective CB1 agonist developed as a treatment against obesity succeeded in clinical trials but had to be withdrawn from the market 3 years later due to its high risk of severe psychiatric disorders including anxiety and suicidal ideations (Christensen et al., 2007; Moreira and Crippa, 2009).

In this line of research, it is imperative to mention the large attention received by a particular cannabinoid substance, CBD. This phytocannabinoid present in *Cannabis sativa* is a natural negative allosteric modular of the CB1 and CB2 receptors. Although its precise mechanism is unclear, CBD has been described to exert neuroprotective and anti-inflammatory effects (Lastres-Becker et al., 2005). Its best described mechanisms of action are: inhibition of FAAH, therefore enhancing AEA signaling (De Petrocellis et al., 2011); and regulation of microglia migration and activation (Martín-Moreno et al., 2011). However, it is important to consider that CBD exhibits more than 65 identified molecular targets across the body (Elsaid and Le Foll, 2020). Such pharmacological complexity makes CBD an interesting candidate for therapeutic research. In fact, CBD has been shown to decrease anxiety-like behavior in animal models of MDD (Hen-Shoval et al., 2018; Xu et al., 2019). In particular, CBD can act *via* CB1 receptors and induce hippocampal neurogenesis associated with anxiolytic effects (Campos et al., 2013; Luján and Valverde, 2020). Similarly, in humans, 62% of self-users of CBD commercial formulations report using this drug in order to treat a medical condition (Corroon and Phillips, 2018). Specifically, consumers are utilizing CBD as a therapy for multiple psychiatric conditions like anxiety and depression, but also pain and sleep disorders. Furthermore, it is well tolerated, non-addictive, and its use is safe (Iffland and Grotenhermen, 2017; World Health Organization and others, 2017). Nevertheless, the efficacy of CBD as a treatment for depression has not been clinically confirmed and remains under investigation. A recent meta-analysis yielding 924 records from clinical trials found that there were no studies investigating the efficacy of CBD by assessing depressive symptoms as the primary outcome (Pinto et al., 2020). Therefore, they concluded that the potential therapeutic properties of CBD as a treatment for MDD needs much further research. In fact, a number of clinical trials are now starting to test CBD as an adjunctive treatment for depressive disorders and, hopefully, the results will be reported in the coming years. Curiously, this year a one case-report described a successful CBD treatment in an adolescent suffering from multiple substance use disorder, social phobia, narcissistic personality disorder, and severe depression (Laczkovics et al., 2020). Researchers reported that the patient was administered CBD upon treatment with antidepressants, after which they showed improvement regarding depressive and anxiety symptoms. Although, this study compromises only one patient, this observation is a quite promising outcome shedding a light on the therapeutic potential of CBD as a treatment for MDD.

Furthermore, in the last 20 years a novel pharmacological treatment has received increasing attention. Ketamine is a chiral compound that displays rapid antidepressant properties after a single intravenous (IV) dose (Murrough et al., 2013). Unfortunately, the exact mechanism by which ketamine exerts its antidepressant effects remains under discussion, although some possible pathways have been proposed. For instance, ketamine seems to inhibit the activation of the HPA-axis signaling via blocking enzyme expression and activity of the adrenal gland (Besnier et al., 2017). Furthermore, ketamine administration has also been linked to increased hippocampal neurogenesis associated to its antidepressant effects (Yamada and Jinno, 2019). Recently, the involvement of the endocannabinoid system in its mechanisms of action has been described (Ferreira et al., 2018; Khakpai et al., 2019). In particular, Ferreira et al. (2018) describes that nociceptive actions of ketamine are connected to AEA release and CB1 activation. These findings reinforce the importance of the ECS in the pathophysiology of MDD and highlight the need for integrative research when studying intricate, heterogenic illnesses like neuropsychiatric disorders.

It is also worth mentioning a novel field of research that has emerged in recent years involving another potential disrupted mechanism contributing to MDD’s pathophysiology. The gut microbiota has been shown to impact a series of physiological mechanisms including cognition and behavior. In fact, a number of microbiome alterations have been correlated to quality of life of MDD patients (Valles-Colomer et al., 2019). However, currently no consensus has emerged about which bacterial strains are more relevant for MDD (Cheung et al., 2019). Importantly, expression of CB1 has also been observed in the gastrointestinal tract (GI), both in the enteric nervous system but also in non-neuronal cells forming the intestinal mucosa (Izzo and Sharkey 2010). Relevant to MDD animal models, Zoppi et al. (2012) reported that CB1 exerted a protective role in the colon of rodents exposed to stress paradigms. Unfortunately, this line of research has not been extensively explored probably due to the complexity of all three mechanisms involved: the ECS, MDD, and the microbiome.

Overall, the synthesis of the above mentioned “puzzle pieces” further supports the claim made throughout this entire paper: the ECS is a very complex system with a variety of functions, which we are only beginning to understand. This, once again, serves as a reminder that narrow and focused research will not be able to uncover its intricate workings, and that of course, interdisciplinary work needs to be prioritized.

## Conclusion

The present review provides a concise overview of the involvement of the ECS in a number of theorized etiologies of MDD such as: genetic factors, hyperactivation of the HPA-axis, dysregulation of immune response mediated by microglia, and loss of neurogenesis. In this manner, epidemiological studies have associated both CB1 and CB2 gene polymorphisms to the development of MDD. Importantly, these associations by themselves are not sufficient to explain the entirety of its pathophysiology due to the essential role played by environmental factors. Furthermore, the ECS is responsible for the termination of HPA-axis signaling after exposure to stress. Particularly, downregulation of CB1 receptors, as a result of prolonged increase of glucocorticoid concentrations induced by chronic stress, is associated to a hyperactive HPA-axis. Hence, endocannabinoid regulation plays an important role in vulnerability to stress, a major risk factor for MDD. Alternatively, CB2 seems to be a primary contributor to the neuroinflammatory etiology of MDD. Evidence supports the role of CB2 in microglia-mediated neuroprotection and anti-inflammatory function. Specifically, AEA-dependent CB2 receptor activation promotes the shift towards immunosuppressive phenotype of microglia. Hence, alterations in CB2 expression, AEA concentrations and FAAH activity could be a trigger of depressive symptomatology. Interestingly, CB1 seems responsible for the communication between HPA-related mechanisms and CB2-mediated microglial activation. Therefore, its contribution to the immune function should not be dismissed. Lastly, neurogenesis is also described as an important mechanism underlying the pathophysiology of MDD. Potential involvement of CB1 as a direct contributor to neurogenetic pathways has been suggested. On the other hand, CB2 seems to be responsible for microglia-induced stimulation and depression of hippocampal cell proliferation. Importantly, HPA-axis activity can also regulate neurogenesis and *vice versa*. This demonstrates the great level of inseparability between the components of the endocannabinoid system and the underlying circuits of major depressive disorder, as well as the need of integrative work that can integrate interdisciplinary findings to achieve a better understanding of heterogenic, multifactorial disorders.

Overall, this review proposes the ECS as a unitary entity of the most important recognized pathways leading to major depressive disorder. It also emphasizes the need for interdisciplinary studies that provide new scopes about its pathophysiology in order to develop more efficient therapies for this devastating disease.

## References

[B1] Aguado T. Palazuelos J. Monory K. Stella N. Cravatt B. Lutz B. (2006). The Endocannabinoid System Promotes Astroglial Differentiation by Acting on Neural Progenitor Cells. J. Neurosci. 26 (5), 1551–1561. 10.1523/JNEUROSCI.3101-05.2006 16452678 PMC6675499

[B2] Ahrens J. Demir R. Leuwer M. de la Roche J. Krampfl K. Foadi N. (2009). The Nonpsychotropic Cannabinoid Cannabidiol Modulates and Directly Activates Alpha-1 and Alpha-1-Beta Glycine Receptor Function. Pharmacology 83 (4), 217–222. 10.1159/000201556 19204413

[B3] Aizpurua-Olaizola O. Soydaner U. Öztürk E. Schibano D. Simsir Y. Navarro P. (2016). Evolution of the Cannabinoid and Terpene Content during the Growth of Cannabis Sativa Plants from Different Chemotypes. J. Nat. Prod. 79 (2), 324–331. 10.1021/acs.jnatprod.5b00949 26836472

[B4] Akil H. Gordon J. Hen R. Javitch J. Mayberg H. McEwen B. (2018). Treatment Resistant Depression: A Multi-Scale, Systems Biology Approach. Neurosci. Biobehav Rev. 84 (January), 272–288. 10.1016/j.neubiorev.2017.08.019 28859997 PMC5729118

[B5] Albert P. (2015). Why Is Depression More Prevalent in Women? J. Psychiatry Neurosci. 40 (4), 219–221. 10.1503/jpn.150205 26107348 PMC4478054

[B6] American Psychiatric Association (2013). Diagnostic and Statistical Manual of Mental Disorders. Fifth Edition. Virginia, United States: American Psychiatric Association. 10.1176/appi.books.9780890425596

[B7] Anacker C. Cattaneo A. Luoni A. Musaelyan K. Zunszain P. A. Milanesi E. (2013). Glucocorticoid-Related Molecular Signaling Pathways Regulating Hippocampal Neurogenesis. Neuropsychopharmacology 38 (5), 872–883. 10.1038/npp.2012.253 23303060 PMC3672002

[B8] Andres-Mach M. Haratym-Maj A. Zagaja M. Rola R. Maj M. Chrościńska-Krawczyk M. (2015). ACEA (A Highly Selective Cannabinoid CB1 Receptor Agonist) Stimulates Hippocampal Neurogenesis in Mice Treated with Antiepileptic Drugs. Brain Res. 1624 (October), 86–94. 10.1016/j.brainres.2015.07.028 26225920

[B9] Anisman H. Merali Z. Hayley S. (2008). Neurotransmitter, Peptide and Cytokine Processes in Relation to Depressive Disorder: Comorbidity between Depression and Neurodegenerative Disorders. Prog. Neurobiol. 85 (1), 1–74. 10.1016/j.pneurobio.2008.01.004 18346832

[B10] Ashton J. C. Friberg D. Darlington C. L. Smith P. F. (2006). Expression of the Cannabinoid CB2 Receptor in the Rat Cerebellum: An Immunohistochemical Study. Neurosci. Lett. 396 (2), 113–116. 10.1016/j.neulet.2005.11.038 16356641

[B11] Aso E. Ozaita A. Valdizán E. M. Ledent C. Pazos A. Maldonado R. (2008). BDNF Impairment in the Hippocampus Is Related to Enhanced Despair Behavior in CB1 Knockout Mice. J. Neurochem. 105 (2), 565–572. 10.1111/j.1471-4159.2007.05149.x 18047561

[B12] Ativie F. Komorowska J. A. Beins E. Albayram Ö. Zimmer T. Zimmer A. (2018). Cannabinoid 1 Receptor Signaling on Hippocampal GABAergic Neurons Influences Microglial Activity. Front. Mol. Neurosci. 11, 295. 10.3389/fnmol.2018.00295 30210289 PMC6121063

[B13] Autry A. E. Adachi M. Cheng P. Monteggia L. M. (2009). Gender-Specific Impact of Brain-Derived Neurotrophic Factor Signaling on Stress-Induced Depression-like Behavior. Biol. Psychiatry 66 (1), 84–90. 10.1016/j.biopsych.2009.02.007 19358977 PMC2734472

[B14] Avraham H. K. Jiang S. Fu Y. Rockenstein E. Makriyannis A. Zvonok A. (2014). The Cannabinoid CB₂ Receptor Agonist AM1241 Enhances Neurogenesis in GFAP/Gp120 Transgenic Mice Displaying Deficits in Neurogenesis. Br. J. Pharmacol. 171 (2), 468–479. 10.1111/bph.12478 24148086 PMC3904265

[B15] Bachmann S. (2018). Epidemiology of Suicide and the Psychiatric Perspective. Int. J. Environ. Res. Public Health 15 (7). 10.3390/ijerph15071425 PMC6068947 29986446

[B16] Bakas T. van Nieuwenhuijzen P. S. Devenish S. O. McGregor I. S. Arnold J. C. Chebib M. (2017). The Direct Actions of Cannabidiol and 2-Arachidonoyl Glycerol at GABAA Receptors. Pharmacol. Res. 119 (May), 358–370. 10.1016/j.phrs.2017.02.022 28249817

[B17] Bambico F. R. Katz N. Debonnel G. Gobbi G. Gobbi Gabriella. (2007). Cannabinoids Elicit Antidepressant-like Behavior and Activate Serotonergic Neurons through the Medial Prefrontal Cortex. J. Neurosci. 27 (43), 11700–11711. 10.1523/JNEUROSCI.1636-07.2007 17959812 PMC6673235

[B18] Barna I. Zelena D. Arszovszki A. C. Ledent C. (2004). The Role of Endogenous Cannabinoids in the Hypothalamo-Pituitary-Adrenal Axis Regulation: *In Vivo* and *In Vitro* Studies in CB1 Receptor Knockout Mice. Life Sci. 75 (24), 2959–2970. 10.1016/j.lfs.2004.06.006 15454346

[B19] Bartel S. J. Sherry S. B. Stewart S. H. (2020). Self-Isolation: A Significant Contributor to Cannabis Use during the COVID-19 Pandemic. Subst. Abus 41 (0), 409–412. 10.1080/08897077.2020.1823550 33044893

[B20] Beins Eva. Carolina. (2020). The Role of the Endocannabinoid System in Stress-Related Disorders and Neuroimmune Communication. Available at: https://bonndoc.ulb.uni-bonn.de/xmlui/handle/20.500.11811/8344 .

[B21] Belle D. Doucet J. (2003). Poverty, Inequality, and Discrimination as Sources of Depression Among U.S. Women. Psychol. Women Q. 27 (2), 101–113. 10.1111/1471-6402.00090

[B22] Benke C. Autenrieth L. K. Asselmann E. Pané-Farré C. A. (2020). Lockdown, Quarantine Measures, and Social Distancing: Associations with Depression, Anxiety and Distress at the Beginning of the COVID-19 Pandemic Among Adults from Germany. Psychiatry Res. 293 (November), 113462. 10.1016/j.psychres.2020.113462 32987222 PMC7500345

[B23] Besnier E. Clavier T. Tonon M. C. Selim J. Lefevre-Scelles A. Morin F. (2017). Ketamine and Etomidate Down-Regulate the Hypothalamic-Pituitary-Adrenal Axis in an Endotoxemic Mouse Model. Anesthesiology 127 (2), 347–354. 10.1097/ALN.0000000000001704 28542000

[B24] Beyer C. E. Dwyer Piesla J. M Piesla M. J. Platt B. J. Shen R. Rahman Z. (2010). Ru Shen, Zia Rahman, Karen Chan, et alDepression-like Phenotype Following Chronic CB1 Receptor Antagonism. Neurobiol. Dis. 39 (2), 148–155. 10.1016/j.nbd.2010.03.020 20381618

[B25] Bond A. M. Ming G. L. Song H. (2015). Adult Mammalian Neural Stem Cells and Neurogenesis: Five Decades Later. Cell Stem Cell 17 (4), 385–395. 10.1016/j.stem.2015.09.003 26431181 PMC4683085

[B26] Bouaboula M. Poinot-Chazel C. Bourrié B. Canat X. Calandra B. Rinaldi-Carmona M. (1995). Activation of Mitogen-Activated Protein Kinases by Stimulation of the Central Cannabinoid Receptor CB1. Biochem. J. 312 ( Pt 2) (Pt 2), 637–641. 10.1042/bj3120637 8526880 PMC1136308

[B27] Brown A. J. (2007). Novel Cannabinoid Receptors. Br. J. Pharmacol. 152 (5), 567–575. 10.1038/sj.bjp.0707481 17906678 PMC2190013

[B28] Busquets-Garcia A. Bains J. Marsicano G. (2018). CB1 Receptor Signaling in the Brain: Extracting Specificity from Ubiquity. Neuropsychopharmacology 43 (1), 4–20. 10.1038/npp.2017.206 28862250 PMC5719111

[B29] Cabral G. A. Raborn E. S. Griffin L. Dennis J. Marciano-Cabral F. (2008). CB2 Receptors in the Brain: Role in Central Immune Function. Br. J. Pharmacol. 153 (2), 240–251. 10.1038/sj.bjp.0707584 18037916 PMC2219530

[B30] Campos A. C. Ortega Z. Palazuelos J. Fogaça M. V. Aguiar D. C. Díaz-Alonso J. (2013). The Anxiolytic Effect of Cannabidiol on Chronically Stressed Mice Depends on Hippocampal Neurogenesis: Involvement of the Endocannabinoid System. Int. J. Neuropsychopharmacol. 16 (6), 1407–1419. 10.1017/S1461145712001502 23298518

[B31] Carlisle S. J. Marciano-Cabral F. Staab A. Ludwick C. Cabral G. A. (2002). Differential Expression of the CB2 Cannabinoid Receptor by Rodent Macrophages and Macrophage-like Cells in Relation to Cell Activation. Int. Immunopharmacol 2 (1), 69–82. 10.1016/s1567-5769(01)00147-3 11789671

[B32] Castillo P. E. Younts T. J. Chávez A. E. Hashimotodani Y. (2012). Endocannabinoid Signaling and Synaptic Function. Neuron 76 (1), 70–81. 10.1016/j.neuron.2012.09.020 23040807 PMC3517813

[B33] Caughey G. E. Mantzioris E. Gibson R. A. Cleland L. G. James M. J. (1996). The Effect on Human Tumor Necrosis Factor Alpha and Interleukin 1 Beta Production of Diets Enriched in N-3 Fatty Acids from Vegetable Oil or Fish Oil. Am. J. Clin. Nutr. 63 (1), 116–122. 10.1093/ajcn/63.1.116 8604658

[B34] Chávez A. E. Chiu C. Q. Castillo P. E. (2010). TRPV1 Activation by Endogenous Anandamide Triggers Postsynaptic Long-Term Depression in Dentate Gyrus. Nat. Neurosci. 13 (12), 1511–1518. 10.1038/nn.2684 21076423 PMC3058928

[B35] ChenCortina H. C. Spiers J. G. Sernia C. Lavidis N. A. (2018). Inhibition of Fatty Acid Amide Hydrolase by PF-3845 Alleviates the Nitrergic and Proinflammatory Response in Rat Hippocampus Following Acute Stress. Int. J. Neuropsychopharmacol. 21 (8), 786–795. 10.1093/ijnp/pyy033 29579222 PMC6070085

[B36] Cheung S. G. GoldenthalGoldenthal A. R. Uhlemann A. C. Mann J. J. Miller J. M. Sublette M. E. (2019). Systematic Review of Gut Microbiota and Major Depression. Front. Psychiatry 10, 34. 10.3389/fpsyt.2019.00034 30804820 PMC6378305

[B37] Christensen R. Kristensen P. K. Bartels E. M. Bliddal H. Astrup A. (2007). Efficacy and Safety of the Weight-Loss Drug Rimonabant: A Meta-Analysis of Randomised Trials. The Lancet 370 (9600), 1706–1713. 10.1016/S0140-6736(07)61721-8 18022033

[B38] Coppen A. (1967). The Biochemistry of Affective Disorders. Br. J. Psychiatry 113 (504), 1237–1264. 10.1192/bjp.113.504.1237 4169954

[B39] Correa F. Hernangómez M. Mestre L. Loría F. Spagnolo A. Docagne F. (2010). Anandamide Enhances IL-10 Production in Activated Microglia by Targeting CB(2) Receptors: Roles of ERK1/2, JNK, and NF-KappaB. Glia 58 (2), 135–147. 10.1002/glia.20907 19565660

[B40] Correa F. Hernangómez-Herrero M. Mestre L. Loría F. Docagne F. Guaza C. (2011). The Endocannabinoid Anandamide Downregulates IL-23 and IL-12 Subunits in a Viral Model of Multiple Sclerosis: Evidence for a Cross-Talk between IL-12p70/IL-23 Axis and IL-10 in Microglial Cells. Brain Behav. Immun. 25 (4), 736–749. 10.1016/j.bbi.2011.01.020 21310228

[B41] Corroon J. Phillips J. A. (2018). A Cross-Sectional Study of Cannabidiol Users. Cannabis Cannabinoid Res. 3 (1), 152–161. 10.1089/can.2018.0006 30014038 PMC6043845

[B42] Costa B. Giagnoni G. Franke C. Trovato A. E. Colleoni M. (2004). Vanilloid TRPV1 Receptor Mediates the Antihyperalgesic Effect of the Nonpsychoactive Cannabinoid, Cannabidiol, in a Rat Model of Acute Inflammation. Br. J. Pharmacol. 143 (2), 247–250. 10.1038/sj.bjp.0705920 15313881 PMC1575333

[B43] Cota D. Steiner M. A. Marsicano G. Cervino C. Herman J. P. Grübler Y. (2007). Requirement of Cannabinoid Receptor Type 1 for the Basal Modulation of Hypothalamic-Pituitary-Adrenal Axis Function. Endocrinology 148 (4), 1574–1581. 10.1210/en.2005-1649 17194743

[B44] Cota D. (2008). The Role of the Endocannabinoid System in the Regulation of Hypothalamic-Pituitary-Adrenal Axis Activity. J. Neuroendocrinol 20 Suppl 1 (Suppl. 1May), 35–38. 10.1111/j.1365-2826.2008.01673.x 18426497

[B45] Cristino L. Bisogno T. Di Marzo V. (2020). Cannabinoids and the Expanded Endocannabinoid System in Neurological Disorders. Nat. Rev. Neurol. 16 (1), 9–29. 10.1038/s41582-019-0284-z 31831863

[B46] D'Souza D. C. Perry E. MacDougall L. Ammerman Y. Cooper T. Wu Y.-t. (2004). The Psychotomimetic Effects of Intravenous Delta-9-Tetrahydrocannabinol in Healthy Individuals: Implications for Psychosis. Neuropsychopharmacol 29 (8), 1558–1572. 10.1038/sj.npp.1300496 15173844

[B187] Dattani S. Ritchie H. Roser M. (2018). Mental Health. Our World in Data. Available at: https://ourworldindata.org/mental-health .

[B47] David D. J. Samuels B. A. Rainer Q. Wang J.-W. Marsteller D. Mendez I. (2009). Neurogenesis-Dependent and -Independent Effects of Fluoxetine in an Animal Model of Anxiety/DepressionBehavioral Effects of Fluoxetine in an Animal Model of Anxiety/Depression Are Mediated by Both Neurogenesis-dependent and Independent Mechanisms. Neuron 62 (4), 479–493. 10.1016/j.neuron.2009.04.017 19477151 PMC2759281

[B48] De Petrocellis L. Ligresti A. Moriello A. S. Allarà M. Bisogno T. Petrosino S. (2011). Effects of Cannabinoids and Cannabinoid-Enriched Cannabis Extracts on TRP Channels and Endocannabinoid Metabolic Enzymes. Br. J. Pharmacol. 163 (7), 1479–1494. 10.1111/j.1476-5381.2010.01166.x 21175579 PMC3165957

[B49] Derkinderen P. Ledent C. Parmentier M. Girault J. A. (2001). Cannabinoids Activate P38 Mitogen-Activated Protein Kinases through CB1 Receptors in Hippocampus. J. Neurochem. 77 (3), 957–960. 10.1046/j.1471-4159.2001.00333.x 11331425

[B50] Devane W. A. Hanus L. Breuer A. Pertwee R. G. Stevenson L. A. Griffin G. (1992). Isolation and Structure of a Brain Constituent that Binds to the Cannabinoid Receptor. Science 258 (5090), 1946–1949. 10.1126/science.1470919 1470919

[B51] Di Marzo V. De Petrocellis L. (2012). Why Do Cannabinoid Receptors Have More Than One Endogenous Ligand? Philos. Trans. R. Soc. Lond. B Biol. Sci. 367 (1607), 3216–3228. 10.1098/rstb.2011.0382 23108541 PMC3481524

[B52] Diana M. A. Marty A. (2004). Endocannabinoid-Mediated Short-Term Synaptic Plasticity: Depolarization-Induced Suppression of Inhibition (DSI) and Depolarization-Induced Suppression of Excitation (DSE). Br. J. Pharmacol. 142 (1), 9–19. 10.1038/sj.bjp.0705726 15100161 PMC1574919

[B53] Dinh T. P. Carpenter D. Leslie F. M. Freund T. F. Katona I. Sensi S. L. (2002). Brain Monoglyceride Lipase Participating in Endocannabinoid Inactivation. Proc. Natl. Acad. Sci. U S A. 99 (16), 10819–10824. 10.1073/pnas.152334899 12136125 PMC125056

[B54] Dinh T. P. Kathuria S. Piomelli D. (2004). RNA Interference Suggests a Primary Role for Monoacylglycerol Lipase in the Degradation of the Endocannabinoid 2-Arachidonoylglycerol. Mol. Pharmacol. 66 (5), 1260–1264. 10.1124/mol.104.002071 15272052

[B55] Domschke K. Dannlowski U. Ohrmann P. Lawford B. Bauer J. Kugel H. (2008). Cannabinoid Receptor 1 (CNR1) Gene: Impact on Antidepressant Treatment Response and Emotion Processing in Major Depression. Eur. Neuropsychopharmacol. 18 (10), 751–759. 10.1016/j.euroneuro.2008.05.003 18579347

[B56] Elsaid S. Le Foll B. (2020). The Complexity of Pharmacology of Cannabidiol (CBD) and its Implications in the Treatment of Brain Disorders. Neuropsychopharmacology 45 (1), 229–230. 10.1038/s41386-019-0518-1 31511618 PMC6879582

[B57] Felger J. C. Haroon E. Patel T. A. Goldsmith D. R. Wommack E. C. Woolwine B. J. (2018). What Does Plasma CRP Tell Us about Peripheral and Central Inflammation in Depression? Mol. Psychiatry 25 (June), 1301–1311. 10.1038/s41380-018-0096-3 29895893 PMC6291384

[B58] Ferreira R. C. M. Castor M. G. M. Piscitelli F. Di Marzo V. Duarte I. D. G. Romero T. R. L. (2018). The Involvement of the Endocannabinoid System in the Peripheral Antinociceptive Action of Ketamine. J. Pain 19 (5), 487–495. 10.1016/j.jpain.2017.12.002 29247851

[B59] Ferrini F. De Koninck Y. (2013). Microglia Control Neuronal Network Excitability via BDNF Signalling. Neural Plast. 2013 (September), 429815. 10.1155/2013/429815 24089642 PMC3780625

[B60] Fogaça M. V. Campos A. C. Coelho L. D. Duman R. S. Guimarães F. S. (2018). The Anxiolytic Effects of Cannabidiol in Chronically Stressed Mice Are Mediated by the Endocannabinoid System: Role of Neurogenesis and Dendritic Remodeling. Neuropharmacology 135, 22–33. 10.1016/j.neuropharm.2018.03.001 29510186

[B61] Fowler C. J. (2015). The Potential of Inhibitors of Endocannabinoid Metabolism as Anxiolytic and Antidepressive Drugs-A Practical View. Eur. Neuropsychopharmacol. 25 (6), 749–762. 10.1016/j.euroneuro.2015.02.005 25791296

[B62] Gaetani S. Dipasquale P. Romano A. Righetti L. Cassano T. Piomelli D. (2009). The Endocannabinoid System as a Target for Novel Anxiolytic and Antidepressant Drugs. Int. Rev. Neurobiol. 85, 57–72. 10.1016/S0074-7742(09)85005-8 19607961

[B63] Gao Y. Vasilyev D. V. Goncalves M. B. Howell F. V. Hobbs C. Reisenberg M. (2010). Loss of Retrograde Endocannabinoid Signaling and Reduced Adult Neurogenesis in Diacylglycerol Lipase Knock-Out Mice. J. Neurosci. 30 (6), 2017–2024. 10.1523/JNEUROSCI.5693-09.2010 20147530 PMC6634037

[B64] García-Gutiérrez M. S. García-Bueno B. Zoppi S. Leza J. C. Manzanares J. Manzanares Jorge. (2012). Chronic Blockade of Cannabinoid CB2 Receptors Induces Anxiolytic-like Actions Associated with Alterations in GABAA Receptors. Br. J. Pharmacol. 165 (4), 951–964. 10.1111/j.1476-5381.2011.01625.x 21838753 PMC3312491

[B65] Gillespie C. F. Nemeroff C. B. (2005). Hypercortisolemia and Depression. Psychosom Med. 67 Suppl 1 (Suppl. 1June), S26–S28. 10.1097/01.psy.0000163456.22154.d2 15953796

[B66] Gonca E. Darıcı F. (2015). The Effect of Cannabidiol on Ischemia/Reperfusion-Induced Ventricular Arrhythmias: The Role of Adenosine A1 Receptors. J. Cardiovasc. Pharmacol. Ther. 20 (1), 76–83. 10.1177/1074248414532013 24853683

[B67] Gonda X. Hullam G. Antal P. Eszlari N. Petschner P. Hökfelt T. G. (2018). Significance of Risk Polymorphisms for Depression Depends on Stress Exposure. Sci. Rep. 8 (1), 3946. 10.1038/s41598-018-22221-z 29500446 PMC5834495

[B68] Graham E. S. Angel C. E. Schwarcz L. E. Dunbar P. R. Glass M. (2010). Detailed Characterisation of CB2 Receptor Protein Expression in Peripheral Blood Immune Cells from Healthy Human Volunteers Using Flow Cytometry. Int. J. Immunopathol Pharmacol. 23 (1), 25–34. 10.1177/039463201002300103 20377992

[B69] Gray J. M. Vecchiarelli H. A. Morena M. Lee T. T. Hermanson D. J. Kim A. B. (2015). Corticotropin-Releasing Hormone Drives Anandamide Hydrolysis in the Amygdala to Promote Anxiety. J. Neurosci. 35 (9), 3879–3892. 10.1523/JNEUROSCI.2737-14.2015 25740517 PMC4348185

[B70] Gray J. M. Wilson C. D. Lee T. T. Pittman Q. J. Deussing J. M. Hillard C. J. (2016). Sustained Glucocorticoid Exposure Recruits Cortico-Limbic CRH Signaling to Modulate Endocannabinoid Function. Psychoneuroendocrinology 66 (April), 151–158. 10.1016/j.psyneuen.2016.01.004 26821211 PMC4788523

[B71] Grueter B. A. Brasnjo G. Malenka R. C. (2010). Postsynaptic TRPV1 Triggers Cell Type-specific Long-Term Depression in the Nucleus Accumbens. Nat. Neurosci. 13 (12), 1519–1525. 10.1038/nn.2685 21076424 PMC3092590

[B72] Gulyas A. I. Cravatt B. F. Bracey M. H. Dinh T. P. Piomelli D. Boscia F. (2004). Segregation of Two Endocannabinoid-Hydrolyzing Enzymes into Pre- and Postsynaptic Compartments in the Rat Hippocampus, Cerebellum and Amygdala. Eur. J. Neurosci. 20 (2), 441–458. 10.1111/j.1460-9568.2004.03428.x 15233753

[B73] Gunduz-CinarHill O. Hill M. N. McEwen B. S. Holmes A. (2013). Amygdala FAAH and Anandamide: Mediating Protection and Recovery from Stress. Trends Pharmacol. Sci. 34 (11), 637–644. 10.1016/j.tips.2013.08.008 24325918 PMC4169112

[B74] Haapakoski R. Mathieu J. Ebmeier K. P. Alenius H. Kivimäki M. (2015). Cumulative Meta-Analysis of Interleukins 6 and 1β, Tumour Necrosis Factor α and C-Reactive Protein in Patients with Major Depressive Disorder. Brain Behav. Immun. 49 (October), 206–215. 10.1016/j.bbi.2015.06.001 26065825 PMC4566946

[B75] Haj-Mirzaian A. Amini-Khoei H. Khoei Amini. Haj-Mirzaian A. Amiri S. Ghesmati M. (2017). Activation of Cannabinoid Receptors Elicits Antidepressant-like Effects in a Mouse Model of Social Isolation Stress. Brain Res. Bull. 130 (April), 200–210. 10.1016/j.brainresbull.2017.01.018 28161196

[B76] Haller J. Bakos N. Szirmay M. Ledent C. Freund T. F. (2002). The Effects of Genetic and Pharmacological Blockade of the CB1 Cannabinoid Receptor on Anxiety. Eur. J. Neurosci. 16 (7), 1395–1398. 10.1046/j.1460-9568.2002.02192.x 12405999

[B77] Han S. Thatte J. Buzard D. J. Jones R. M. (2013). Therapeutic Utility of Cannabinoid Receptor Type 2 (CB(2)) Selective Agonists. J. Med. Chem. 56 (21), 8224–8256. 10.1021/jm4005626 23865723

[B78] Hanson N. D. Owens M. J. Nemeroff C. B. (2011). Depression, Antidepressants, and Neurogenesis: A Critical Reappraisal. Neuropsychopharmacology 36 (13), 2589–2602. 10.1038/npp.2011.220 21937982 PMC3230505

[B79] Hashioka S. Klegeris A. Monji A. Kato T. Sawada M. McGeer P. L. (2007). Antidepressants Inhibit Interferon-Gamma-Induced Microglial Production of IL-6 and Nitric Oxide. Exp. Neurol. 206 (1), 33–42. 10.1016/j.expneurol.2007.03.022 17481608

[B80] Heim C. Nemeroff C. B. (2002). Neurobiology of Early Life Stress: Clinical Studies. Semin. Clin. Neuropsychiatry 7 (2), 147–159. 10.1053/scnp.2002.33127 11953939

[B81] Hen-Shoval D. Amar S. Shbiro L. Smoum R. Haj C. G. Mechoulam R. (2018). Acute Oral Cannabidiolic Acid Methyl Ester Reduces Depression-like Behavior in Two Genetic Animal Models of Depression. Behav. Brain Res. 351 (October), 1–3. 10.1016/j.bbr.2018.05.027 29860002

[B82] Hill M. N. Gorzalka B. B. (2005). Is There a Role for the Endocannabinoid System in the Etiology and Treatment of Melancholic Depression? Behav. Pharmacol. 16 (5–6), 333–352. 10.1097/00008877-200509000-00006 16148438

[B83] Hill M. N. Gorzalka B. B. (2005). Pharmacological Enhancement of Cannabinoid CB1 Receptor Activity Elicits an Antidepressant-like Response in the Rat Forced Swim Test. Eur. Neuropsychopharmacol. 15 (6), 593–599. 10.1016/j.euroneuro.2005.03.003 15916883

[B84] Hill M. N. HoHillard W. S. Hillard C. J. Gorzalka B. B. (2008).Differential Effects of the Antidepressants Tranylcypromine and Fluoxetine on Limbic Cannabinoid Receptor Binding and Endocannabinoid Contents. J. Neural Transm. (Vienna) 115, 1673–1679. 10.1007/s00702-008-0131-7 18974922 PMC2992975

[B85] Hill M. N. McLaughlin R. J. Bin. Pan. Karatsoreos B. Christopher Fitzgerald M. L. Roberts C. J. Lee T. T. (2011). Recruitment of Prefrontal Cortical Endocannabinoid Signaling by Glucocorticoids Contributes to Termination of the Stress Response. J. Neurosci. 31 (29), 10506–10515. 10.1523/JNEUROSCI.0496-11.2011 21775596 PMC3179266

[B86] Hill M. N. Miller G. E. Ho W. S. Gorzalka B. B. Hillard C. J. (2008). Serum Endocannabinoid Content Is Altered in Females with Depressive Disorders: A Preliminary Report. Pharmacopsychiatry 41 (2), 48–53. 10.1055/s-2007-993211 18311684 PMC3422568

[B87] Hillard C. J. Beatka M. Sarvaideo. J. (2016). Endocannabinoid Signaling and the Hypothalamic-Pituitary-Adrenal Axis. Compr. Physiol. 7 (1), 1–15. 10.1002/cphy.c160005 28134998 PMC5871916

[B88] Hinwood M. Tynan Ross. J. Tynan J. L. Beynon S. B. Day T. A. Walker F. R. (20131991). Chronic Stress Induced Remodeling of the Prefrontal Cortex: Structural Re-organization of Microglia and the Inhibitory Effect of Minocycline. Cereb. Cortex 23 (8), 1784–1797. 10.1093/cercor/bhs151 22710611

[B89] Horowitz M. A. Wertz J. Zhu D. Cattaneo A. Musaelyan K. Nikkheslat N. (2015). Antidepressant Compounds Can Be Both Pro- and Anti-inflammatory in Human Hippocampal Cells. Int. J. Neuropsychopharmacol. 18 (3). 10.1093/ijnp/pyu076 PMC4360247 25522414

[B90] Howlett A. C. Barth F. Bonner T. I. Cabral G. Casellas P. Devane W. A. (2002). International Union of Pharmacology. XXVII. Classification of Cannabinoid Receptors. Pharmacol. Rev. 54 (2), 161–202. 10.1124/pr.54.2.161 12037135

[B91] Hutch C. R. Hegg C. C. (2016). Cannabinoid Receptor Signaling Induces Proliferation but Not Neurogenesis in the Mouse Olfactory Epithelium. Neurogenesis (Austin) 3 (1), e1118177. 10.1080/23262133.2015.1118177 27606334 PMC4973592

[B92] Icick R. Peoc'h K. Karsinti E. Ksouda K. Hajj A. Bloch V. (2015). A Cannabinoid Receptor 1 Polymorphism Is Protective against Major Depressive Disorder in Methadone-Maintained Outpatients. Am. J. Addict. 24 (7), 613–620. 10.1111/ajad.12273 26331953

[B93] Iffland K. Grotenhermen F. (2017). An Update on Safety and Side Effects of Cannabidiol: A Review of Clinical Data and Relevant Animal Studies. Cannabis Cannabinoid Res. 2 (1), 139–154. 10.1089/can.2016.0034 28861514 PMC5569602

[B94] Izzo A. A. Sharkey K. A. (2010). Cannabinoids and the Gut: New Developments and Emerging Concepts. Pharmacol. Ther. 126 (1), 21–38. 10.1016/j.pharmthera.2009.12.005 20117132

[B95] Jayatissa M. N. Henningsen K. Nikolajsen G. West M. J. Wiborg O. (2010). A Reduced Number of Hippocampal Granule Cells Does Not Associate with an Anhedonia-like Phenotype in a Rat Chronic Mild Stress Model of Depression. Stress 13 (2), 95–105. 10.3109/10253890902951786 19929309

[B96] Jenniches I. Ternes S. Albayram O. Otte D. M. Bach K. Bindila L. (2016). Anxiety, Stress, and Fear Response in Mice with Reduced Endocannabinoid Levels. Biol. Psychiatry 79 (10), 858–868. 10.1016/j.biopsych.2015.03.033 25981172

[B97] Jesulola E. Micalos P. Baguley I. J. (2018). Understanding the Pathophysiology of Depression: From Monoamines to the Neurogenesis Hypothesis Model - Are We There yet? Behav. Brain Res. 341 (April), 79–90. 10.1016/j.bbr.2017.12.025 29284108

[B98] Jin X. H. Okamoto Y. Morishita J. Tsuboi K. Tonai T. Ueda N. (2007). Discovery and Characterization of a Ca2+-independent Phosphatidylethanolamine N-Acyltransferase Generating the Anandamide Precursor and its Congeners. J. Biol. Chem. 282 (6), 3614–3623. 10.1074/jbc.M606369200 17158102

[B99] Jin X. H. Uyama T. Wang J. Okamoto Y. Tonai T. Ueda N. (2009). cDNA Cloning and Characterization of Human and Mouse Ca(2+)-independent Phosphatidylethanolamine N-Acyltransferases. Biochim. Biophys. Acta 1791 (1), 32–38. 10.1016/j.bbalip.2008.09.006 19000777

[B100] Juruena M. F. (2014). Early-Life Stress and HPA Axis Trigger Recurrent Adulthood Depression. Epilepsy Behav. 38 (September), 148–159. 10.1016/j.yebeh.2013.10.020 24269030

[B101] Kaplan H. B. Martin S. S. Johnson R. J. Robbins C. A. (1986). Escalation of Marijuana Use: Application of a General Theory of Deviant Behavior. J. Health Soc. Behav. 27 (1), 44–61. 10.2307/2136502 3486897

[B102] Kaur R. Sidhu P. Singh S. (2016). What Failed BIA 10-2474 Phase I Clinical Trial? Global Speculations and Recommendations for Future Phase I Trials. J. Pharmacol. Pharmacother. 7 (3), 120–126. 10.4103/0976-500X.189661 27651707 PMC5020770

[B103] Kellogg R. Mackie K. Straiker A. (2009). Cannabinoid CB1 Receptor-dependent Long-Term Depression in Autaptic Excitatory Neurons. J. Neurophysiol. 102 (2), 1160–1171. 10.1152/jn.00266.2009 19494194 PMC2724344

[B104] Kendler K. S. Gatz M. Gardner C. O. Pedersen N. L. (2006). A Swedish National Twin Study of Lifetime Major Depression. Am. J. Psychiatry 163 (1), 109–114. 10.1176/appi.ajp.163.1.109 16390897

[B105] Khakpai F. Ebrahimi-Ghiri M. Alijanpour S. Zarrindast M. R. (2019). Ketamine-Induced Antidepressant like Effects in Mice: A Possible Involvement of Cannabinoid System. Biomed. Pharmacother. 112 (April), 108717. 10.1016/j.biopha.2019.108717 30970516

[B106] Kim J. Alger B. E. (2004). Inhibition of Cyclooxygenase-2 Potentiates Retrograde Endocannabinoid Effects in Hippocampus. Nat. Neurosci. 7 (7), 697–698. 10.1038/nn1262 15184902

[B107] Kim J. Alger B. E. (2010). Reduction in Endocannabinoid Tone Is a Homeostatic Mechanism for Specific Inhibitory Synapses. Nat. Neurosci. 13 (5), 592–600. 10.1038/nn.2517 20348918 PMC2860695

[B108] Klegeris A. Bissonnette C. J. McGeer P. L. (2003). Reduction of Human Monocytic Cell Neurotoxicity and Cytokine Secretion by Ligands of the Cannabinoid-type CB2 Receptor. Br. J. Pharmacol. 139 (4), 775–786. 10.1038/sj.bjp.0705304 12813001 PMC1573900

[B109] Koscsó B. Csóka B. Kókai E. Németh Z. H. Pacher P. Virág L. (2013). Adenosine Augments IL‐10‐induced STAT3 Signaling in M2c Macrophages. J. Leukoc. Biol. 94 (6), 1309–1315. 10.1189/jlb.0113043 23922379 PMC3828607

[B110] Kreisel T. Frank M. G. Licht T. Reshef R. Ben-Menachem-Zidon O. Baratta M. V. (2014). Dynamic Microglial Alterations Underlie Stress-Induced Depressive-like Behavior and Suppressed Neurogenesis. Mol. Psychiatry 19 (6), 699–709. 10.1038/mp.2013.155 24342992

[B111] Kubitz N. Mehra M. Potluri R. C. Garg N. Cossrow N. (2013). Characterization of Treatment Resistant Depression Episodes in a Cohort of Patients from a US Commercial Claims Database. PLOS ONE 8 (10), e76882. 10.1371/journal.pone.0076882 24204694 PMC3799999

[B112] Laczkovics C. Kothgassner O. D. Felnhofer A. Klier C. M. (2020). Cannabidiol Treatment in an Adolescent with Multiple Substance Abuse, Social Anxiety and Depression. Neuropsychiatr, 35, 31–34. 10.1007/s40211-020-00334-0 32052321 PMC7954719

[B113] Laprairie R. B. Bagher A. M. Kelly M. E. Denovan-Wright E. M. (2015). Cannabidiol Is a Negative Allosteric Modulator of the Cannabinoid CB1 Receptor. Br. J. Pharmacol. 172 (20), 4790–4805. 10.1111/bph.13250 26218440 PMC4621983

[B114] Lastres-Becker I. Molina-Holgado Francisco. Molina-Holgado F. Ramos J. A. Mechoulam R. Fernández-Ruiz J. (2005). Cannabinoids Provide Neuroprotection against 6-hydroxydopamine Toxicity *In Vivo* and *In Vitro*: Relevance to Parkinson's Disease. Neurobiol. Dis. 19 (1), 96–107. 10.1016/j.nbd.2004.11.009 15837565

[B115] Lazary J. Eszlari N. Juhasz G. Bagdy G. (2019). A Functional Variant of CB2 Receptor Gene Interacts with Childhood Trauma and FAAH Gene on Anxious and Depressive Phenotypes. J. Affect Disord. 257 (October), 716–722. 10.1016/j.jad.2019.07.083 31382124

[B116] Lazary J. Eszlari N. Juhasz G. Bagdy G. (2016). Genetically Reduced FAAH Activity May Be a Risk for the Development of Anxiety and Depression in Persons with Repetitive Childhood Trauma. Eur. Neuropsychopharmacol. 26 (6), 1020–1028. 10.1016/j.euroneuro.2016.03.003 27005594

[B117] Ledent C. Valverde O. Cossu G. Petitet F. Aubert J. F. Beslot F. (1999). Unresponsiveness to Cannabinoids and Reduced Addictive Effects of Opiates in CB1 Receptor Knockout Mice. Science 283 (5400), 401–404. 10.1126/science.283.5400.401 9888857

[B118] Lee S. H. Ledri M. Tóth B. Marchionni I. Henstridge C. M. Dudok B. (2015). Multiple Forms of Endocannabinoid and Endovanilloid Signaling Regulate the Tonic Control of GABA Release. J. Neurosci. 35 (27), 10039–10057. 10.1523/JNEUROSCI.4112-14.2015 26157003 PMC4495235

[B119] Lee T. T. Y. Hill M. N. (2013). Age of Stress Exposure Modulates the Immediate and Sustained Effects of Repeated Stress on Corticolimbic Cannabinoid CB1 Receptor Binding in Male Rats. Neuroscience 249 (September), 106–114. 10.1016/j.neuroscience.2012.11.017 23200786

[B120] Liao Y. Xie B. Zhang H. He Q. Guo L. Subramaniapillai M. (2019). Efficacy of Omega-3 PUFAs in Depression: A Meta-Analysis. Transl Psychiatry 9 (1), 190–199. 10.1038/s41398-019-0515-5 31383846 PMC6683166

[B121] Lisboa S. F. Niraula A. Resstel L. B. Guimaraes F. S. Godbout J. P. Sheridan J. F. (2018). Repeated Social Defeat-Induced Neuroinflammation, Anxiety-like Behavior and Resistance to Fear Extinction Were Attenuated by the Cannabinoid Receptor Agonist WIN55,212-2. Neuropsychopharmacology 43 (9), 1924–1933. 10.1038/s41386-018-0064-2 29786066 PMC6046035

[B122] Liu L.-L. Li J.-M. Su W.-J. Wang B. Jiang C.-L. (2019). Sex Differences in Depressive-like Behaviour May Relate to Imbalance of Microglia Activation in the Hippocampus. Brain Behav. Immun. 81 (October), 188–197. 10.1016/j.bbi.2019.06.012 31181346

[B123] Lively S. Schlichter L. C. (2018). Microglia Responses to Pro-inflammatory Stimuli (LPS, IFNγ+TNFα) and Reprogramming by Resolving Cytokines (IL-4, IL-10). Front Cel Neurosci 12, 215. 10.3389/fncel.2018.00215 PMC6066613 30087595

[B124] Long J. Z. NomuraNomura, Robert E. VannWalentiny D. K. D. Matthew. Vann R. E. Walentiny D. M. Booker L. Jin X. (2009). Dual Blockade of FAAH and MAGL Identifies Behavioral Processes Regulated by Endocannabinoid Crosstalk *In Vivo* . Proc. Natl. Acad. Sci. U S A. 106 (48), 20270–20275. 10.1073/pnas.0909411106 19918051 PMC2787168

[B125] Lopez-Munoz F. Alamo C. (2009). Monoaminergic Neurotransmission: The History of the Discovery of Antidepressants from 1950s until Today. Cpd 15 (14), 1563–1586. 10.2174/138161209788168001 19442174

[B126] Luján M. Á. Valverde. O. (2020). The Pro-neurogenic Effects of Cannabidiol and its Potential Therapeutic Implications in Psychiatric Disorders. Front. Behav. Neurosci. 14 (June), 109. 10.3389/fnbeh.2020.00109 32676014 PMC7333542

[B127] Luo X. Q. Li A. Yang X. Xiao X. Hu R. Wang T. W. (2018). Paeoniflorin Exerts Neuroprotective Effects by Modulating the M1/M2 Subset Polarization of Microglia/Macrophages in the Hippocampal CA1 Region of Vascular Dementia Rats via Cannabinoid Receptor 2. Chin. Med. 13 (1), 14. 10.1186/s13020-018-0173-1 29560022 PMC5859430

[B128] Luo Y. Kataoka Y. Ostinelli E. G. Cipriani A. Furukawa T. A. (2020). National Prescription Patterns of Antidepressants in the Treatment of Adults with Major Depression in the US between 1996 and 2015: A Population Representative Survey Based Analysis. Front. Psychiatry 11. 10.3389/fpsyt.2020.00035 PMC7033625 32116850

[B129] Ma Y. Wang J. Wang Y. Yang G. Y. (2017). The Biphasic Function of Microglia in Ischemic Stroke. Prog. Neurobiol. 157 (October), 247–272. 10.1016/j.pneurobio.2016.01.005 26851161

[B130] Maccarrone M. Rossi S. Bari M. De Chiara V. Fezza F. Musella A. (2008). Anandamide Inhibits Metabolism and Physiological Actions of 2-Arachidonoylglycerol in the Striatum. Nat. Neurosci. 11 (2), 152–159. 10.1038/nn2042 18204441

[B131] Mackie K. (2005). Distribution of Cannabinoid Receptors in the Central and Peripheral Nervous System. Handb Exp. Pharmacol. 168, 299–325. 10.1007/3-540-26573-2_10 16596779

[B132] Maejima T. Hashimoto K. Yoshida T. Aiba A. Kano M. (2001). Presynaptic Inhibition Caused by Retrograde Signal from Metabotropic Glutamate to Cannabinoid Receptors. Neuron 31 (3), 463–475. 10.1016/s0896-6273(01)00375-0 11516402

[B133] Malan T. P. Ibrahim M. M. Deng H. Liu Q. Mata H. P. Vanderah T. (2001). CB2 Cannabinoid Receptor-Mediated Peripheral Antinociception. Pain 93 (3), 239–245. 10.1016/s0304-3959(01)00321-9 11514083

[B134] Malberg J. E. Duman R. S. (2003). Cell Proliferation in Adult Hippocampus Is Decreased by Inescapable Stress: Reversal by Fluoxetine Treatment. Neuropsychopharmacology 28 (9), 1562–1571. 10.1038/sj.npp.1300234 12838272

[B135] Malek N. Popiolek-Barczyk K. Mika J. Przewlocka B. Starowicz K. (20152015). Anandamide, Acting viaCB2Receptors, Alleviates LPS-Induced Neuroinflammation in Rat Primary Microglial Cultures. Neural Plasticity 2015, 1–10. 10.1155/2015/130639 PMC4452105 26090232

[B136] Mammana S. Cavalli E. Gugliandolo A. Silvestro S. Pollastro F. Bramanti P. (2019). Could the Combination of Two Non-psychotropic Cannabinoids Counteract Neuroinflammation? Effectiveness of Cannabidiol Associated with Cannabigerol. Medicina (Kaunas) 55 (11), 747. 10.3390/medicina55110747 PMC6915685 31752240

[B137] Manich G. Recasens M. Valente T. Almolda B. González B. Castellano B. (2019). Role of the CD200-Cd200r Axis during Homeostasis and Neuroinflammation. Neuroscience 405 (May), 118–136. 10.1016/j.neuroscience.2018.10.030 30367946

[B138] Marangell L. B. Martinez J. M. Zboyan H. A. Kertz B. Kim H. F. Puryear L. J. (2003). A Double-Blind, Placebo-Controlled Study of the Omega-3 Fatty Acid Docosahexaenoic Acid in the Treatment of Major Depression. Am. J. Psychiatry 160 (5), 996–998. 10.1176/appi.ajp.160.5.996 12727707

[B139] Marco E. M. Ballesta J. A. Irala C. Hernández M.-D. Serrano M. E. Mela V. (2017). Javier Antonio Ballesta, Carlos Irala, María-Donina Hernández, María Elisa Serrano, Virginia Mela, Meritxell López-Gallardo, and María-Paz ViverosSex-dependent Influence of Chronic Mild Stress (CMS) on Voluntary Alcohol Consumption; Study of Neurobiological Consequences. Pharmacol. Biochem. Behav. 152 (January), 68–80. 10.1016/j.pbb.2016.11.005 27894930

[B140] Martin M. Ledent C. Parmentier M. Maldonado R. Valverde O. (2002). Involvement of CB1 Cannabinoid Receptors in Emotional Behaviour. Psychopharmacology (Berl) 159 (4), 379–387. 10.1007/s00213-001-0946-5 11823890

[B141] Martín-Moreno A. M. Reigada D. Ramírez B. G. Mechoulam R. Innamorato N. Cuadrado A. (2011). Cannabidiol and Other Cannabinoids Reduce Microglial Activation *In Vitro* and *In Vivo*: Relevance to Alzheimer's Disease. Mol. Pharmacol. 79 (6), 964–973. 10.1124/mol.111.071290 21350020 PMC3102548

[B142] Massart R. Mongeau R. Lanfumey L. (2012). Beyond the Monoaminergic Hypothesis: Neuroplasticity and Epigenetic Changes in a Transgenic Mouse Model of Depression. Philos. Trans. R. Soc. Lond. B Biol. Sci. 367 (1601), 2485–2494. 10.1098/rstb.2012.0212 22826347 PMC3405682

[B143] Matias I. Pochard P. Orlando P. Salzet M. Pestel J. Di Marzo V. (2002). Presence and Regulation of the Endocannabinoid System in Human Dendritic Cells. Eur. J. Biochem. 269 (15), 3771–3778. 10.1046/j.1432-1033.2002.03078.x 12153574

[B144] Matsuda L. A. Lolait S. J. Brownstein M. J. Young A. C. Bonner T. I. (1990). Structure of a Cannabinoid Receptor and Functional Expression of the Cloned CDNA. Nature 346 (6284), 561–564. 10.1038/346561a0 2165569

[B145] McKinney M. K. Cravatt B. F. (2005). Structure and Function of Fatty Acid Amide Hydrolase. Annu. Rev. Biochem. 74, 411–432. 10.1146/annurev.biochem.74.082803.133450 15952893

[B146] Mechoulam R. Parker L. A. (2013). The Endocannabinoid System and the Brain. Annu. Rev. Psychol. 64 (1), 21–47. 10.1146/annurev-psych-113011-143739 22804774

[B147] Mensching L. Djogo N. Keller C. Rading S. Karsak M. (2019). Stable Adult Hippocampal Neurogenesis in Cannabinoid Receptor CB2 Deficient Mice. Int. J. Mol. Sci. 20 (15), 3759. 10.3390/ijms20153759 PMC6696320 31374821

[B148] Micale V. Drago F. (2018). Endocannabinoid System, Stress and HPA Axis. Eur. J. Pharmacol. 834 (September), 230–239. 10.1016/j.ejphar.2018.07.039 30036537

[B149] Mitchell P. B. Morris. M. J. (2007). Depression and Anxiety with Rimonabant. Lancet 370 (9600), 1671–1672. 10.1016/S0140-6736(07)61705-X 18022023

[B150] Mitjans M. Serretti A. Fabbri C. Gastó C. Catalán R. Fañanás L. (2013). Screening Genetic Variability at the CNR1 Gene in Both Major Depression Etiology and Clinical Response to Citalopram Treatment. Psychopharmacology (Berl) 227 (3), 509–519. 10.1007/s00213-013-2995-y 23407780

[B151] Mondimore F. M. Zandi P. P. Mackinnon D. F. McInnis Melvin. G. Erin B. Miller M. G. (2006). Familial Aggregation of Illness Chronicity in Recurrent, Early-Onset Major Depression Pedigrees. Am. J. Psychiatry 163 (9), 1554–1560. 10.1176/ajp.2006.163.9.1554 16946180

[B152] Moreira F. A. Grieb M. Lutz. B. (2009). Central Side-Effects of Therapies Based on CB1 Cannabinoid Receptor Agonists and Antagonists: Focus on Anxiety and Depression. Best Pract. Res. Clin. Endocrinol. Metab. 23 (1), 133–144. 10.1016/j.beem.2008.09.003 19285266

[B153] Moreira F. A. CrippaCrippa J. A. S. (2009). The Psychiatric Side-Effects of Rimonabant. Rev. Bras. Psiquiatr. 31 (2), 145–153. 10.1590/s1516-44462009000200012 19578688

[B154] Morin C. M. Carrier J. Bastien C. Godbout R. (2020). Sleep and Circadian Rhythm in Response to the COVID-19 Pandemic. Can. J. Public Health 111, 654–657. 10.17269/s41997-020-00382-7 32700231 PMC7375451

[B155] Mozaffari-Khosravi H. Yassini-Ardakani M. Karamati M. Shariati-Bafghi S.-E. (2013). Eicosapentaenoic Acid versus Docosahexaenoic Acid in Mild-To-Moderate Depression: A Randomized, Double-Blind, Placebo-Controlled Trial. Eur. Neuropsychopharmacol. 23 (7), 636–644. 10.1016/j.euroneuro.2012.08.003 22910528

[B156] Munro S. Thomas K. L. Abu-Shaar M. (1993). Molecular Characterization of a Peripheral Receptor for Cannabinoids. Nature 365 (6441), 61–65. 10.1038/365061a0 7689702

[B157] Murataeva N. Straiker A. Mackie K. (2014). Parsing the Players: 2-Arachidonoylglycerol Synthesis and Degradation in the CNS. Br. J. Pharmacol. 171 (6), 1379–1391. 10.1111/bph.12411 24102242 PMC3954479

[B158] Murillo-Rodriguez E. Poot-Ake A. Arias-Carrion O. Pacheco-Pantoja E. de la Fuente-Ortegon A. Arankowsky-Sandoval G. (2011). The Emerging Role of the Endocannabinoid System in the Sleep-Wake Cycle Modulation. Cnsamc 11 (3), 189–196. 10.2174/187152411798047780 21919868

[B159] Murrough J. W. Iosifescu D. V. Chang L. C. Rayan Al Jurdi R. K. Andrew Green C. E. Perez A. M. (2013). Antidepressant Efficacy of Ketamine in Treatment-Resistant Major Depression: A Two-Site Randomized Controlled Trial. Am. J. Psychiatry 170 (10), 1134–1142. 10.1176/appi.ajp.2013.13030392 23982301 PMC3992936

[B160] Musella A. De Chiara V. Rossi S. Prosperetti C. Bernardi G. Maccarrone M. (2009). TRPV1 Channels Facilitate Glutamate Transmission in the Striatum. Mol. Cel Neurosci 40 (1), 89–97. 10.1016/j.mcn.2008.09.001 18930149

[B161] Nair A. Bonneau R. H. (2006). Stress-Induced Elevation of Glucocorticoids Increases Microglia Proliferation through NMDA Receptor Activation. J. Neuroimmunol 171 (1), 72–85. 10.1016/j.jneuroim.2005.09.012 16278020

[B162] National Collaborating Centre for Mental Health (Uk) (2010) Depression: The Treatment and Management of Depression in Adults. in National Institute for Health and Clinical Excellence: Guidance. Updated Edition (Leicester (UK): British Psychological Society). 22132433

[B163] Navarrete F. Pérez-OrtizPérez-Ortiz J. M. Manzanares J. (2012). Cannabinoid CB₂ Receptor-Mediated Regulation of Impulsive-like Behaviour in DBA/2 Mice. Br. J. Pharmacol. 165 (1), 260–273. 10.1111/j.1476-5381.2011.01542.x 21671903 PMC3252982

[B164] Navarrete M. Araque A. (2008). Endocannabinoids Mediate Neuron-Astrocyte Communication. Neuron 57 (6), 883–893. 10.1016/j.neuron.2008.01.029 18367089

[B165] Navarria A. Tamburella A. Iannotti F. A. Micale V. Camillieri G. Gozzo L. (2014). The Dual Blocker of FAAH/TRPV1 N-Arachidonoylserotonin Reverses the Behavioral Despair Induced by Stress in Rats and Modulates the HPA-Axis. Pharmacol. Res. 87 (September), 151–159. 10.1016/j.phrs.2014.04.014 24861565

[B166] Nelson C. A. Gabard-Durnam L. J. (2020). Early Adversity and Critical Periods: Neurodevelopmental Consequences of Violating the Expectable Environment. Trends Neurosci. 43 (3), 133–143. 10.1016/j.tins.2020.01.002 32101708 PMC8092448

[B167] Nemeroff Charles. B. (2007). Prevalence and Management of Treatment-Resistant Depression. J. Clin. Psychiatry 68 (Suppl. 8), 17–25. 17640154

[B168] Nestler E. J. Barrot M. DiLeone R. J. Eisch A. J. Gold S. J. Monteggia L. M. (2002). Neurobiology of Depression. Neuron 34 (1), 13–25. 10.1016/s0896-6273(02)00653-0 11931738

[B169] Okamoto Y. Morishita J. Tsuboi K. Tonai T. Ueda N. (2004). Molecular Characterization of a Phospholipase D Generating Anandamide and its Congeners. J. Biol. Chem. 279 (7), 5298–5305. 10.1074/jbc.M306642200 14634025

[B170] Onaivi E. S. Ishiguro H. Gong J. P. Patel S. Meozzi P. A. Myers L. (2008). Brain Neuronal CB2 Cannabinoid Receptors in Drug Abuse and Depression: From Mice to Human Subjects. PLoS One 3 (2), e1640. 10.1371/journal.pone.0001640 18286196 PMC2241668

[B171] Pacher P. Bátkai S. Kunos G. (2006). The Endocannabinoid System as an Emerging Target of Pharmacotherapy. Pharmacol. Rev. 58 (3), 389–462. 10.1124/pr.58.3.2 16968947 PMC2241751

[B172] Palazuelos J. Aguado T. Egia A. Mechoulam R. Guzmán M. Galve-Roperh I. (2006). Non-Psychoactive CB2 Cannabinoid Agonists Stimulate Neural Progenitor Proliferation. FASEB J. 20 (13), 2405–2407. 10.1096/fj.06-6164fje 17015409

[B173] Panikashvili D. Mechoulam R. Beni S. M. Alexandrovich A. Shohami E. (2005). CB1 Cannabinoid Receptors Are Involved in Neuroprotection via NF-Kappa B Inhibition. J. Cereb. Blood Flow Metab. 25 (4), 477–484. 10.1038/sj.jcbfm.9600047 15729296

[B174] Patel S. Hillard C. J. (2006). Pharmacological Evaluation of Cannabinoid Receptor Ligands in a Mouse Model of Anxiety: Further Evidence for an Anxiolytic Role for Endogenous Cannabinoid Signaling. J. Pharmacol. Exp. Ther. 318 (1), 304–311. 10.1124/jpet.106.101287 16569753

[B175] Patel S. Hillard C. J. (2009). Role of Endocannabinoid Signaling in Anxiety and Depression. Curr. Top. Behav. Neurosci. 1, 347–371. 10.1007/978-3-540-88955-7_14 21104391 PMC3808114

[B176] Patel S. Roelke C. T. Rademacher D. J. Hillard C. J. (2005). Inhibition of Restraint Stress-Induced Neural and Behavioural Activation by Endogenous Cannabinoid Signalling. Eur. J. Neurosci. 21 (4), 1057–1069. 10.1111/j.1460-9568.2005.03916.x 15787710

[B177] Pertwee R. G. Howlett A. C. Abood M. E. Alexander S. P. Di Marzo V. Elphick M. R. (2010). International Union of Basic and Clinical Pharmacology. LXXIX. Cannabinoid Receptors and Their Ligands: beyond CB₁ and CB₂. Pharmacol. Rev. 62 (4), 588–631. 10.1124/pr.110.003004 21079038 PMC2993256

[B178] Pertwee R. G. Roger G. (2006). Cannabinoid Pharmacology: The First 66 Years. Br. J. Pharmacol. 147 Suppl 1 (Suppl. 1), S163–S171. 10.1038/sj.bjp.0706406 16402100 PMC1760722

[B179] Pertwee R. G. (2008). The Diverse CB1 and CB2 Receptor Pharmacology of Three Plant Cannabinoids: delta9-tetrahydrocannabinol, Cannabidiol and delta9-tetrahydrocannabivarin. Br. J. Pharmacol. 153 (2), 199–215. 10.1038/sj.bjp.0707442 17828291 PMC2219532

[B180] Phan K. L. Angstadt M. Golden J. Onyewuenyi I. Popovska A. de Wit H. (2008). Mike Angstadt, Jamie Golden, Ikechukwu Onyewuenyi, Ana Popovska, and Harriet de WitCannabinoid Modulation of Amygdala Reactivity to Social Signals of Threat in Humans. J. Neurosci. 28 (10), 2313–2319. 10.1523/JNEUROSCI.5603-07.2008 18322078 PMC2657360

[B181] Pinto J. V. Saraf G. Frysch C. Vigo D. Keramatian K. Chakrabarty T. (2020). Cannabidiol as a Treatment for Mood Disorders: A Systematic Review: Le cannabidiol comme traitement des troubles de l'humeur: une revue systématique. Can. J. Psychiatry 65 (4), 213–227. 10.1177/0706743719895195 31830820 PMC7385425

[B182] Pratt L. A. Brody D. J. (2008). Depression in the United States Household Population, 2005-2006. NCHS Data Brief 18 (September)), 1–8. 19389321

[B183] Rai D. Zitko P. Jones K. Lynch J. Araya R. (2013). Country- and Individual-Level Socioeconomic Determinants of Depression: Multilevel Cross-National Comparison. Br. J. Psychiatry 202 (3), 195–203. 10.1192/bjp.bp.112.112482 23349294

[B184] Ramirez S. H. Reichenbach N. L. Fan S. Rom S. Merkel S. F. Wang X. (2013). Attenuation of HIV-1 Replication in Macrophages by Cannabinoid Receptor 2 Agonists. J. Leukoc. Biol. 93 (5), 801–810. 10.1189/jlb.1012523 23463725 PMC3629438

[B185] Ransohoff R. M. (2016). A Polarizing Question: Do M1 and M2 Microglia Exist? Nat. Neurosci. 19 (8), 987–991. 10.1038/nn.4338 27459405

[B186] Major Depressive Disorder Working Group of the Psychiatric GWAS Consortium Ripke Stephan. Ripke S. WrayLewis N. R. Steven. P. Hamilton. Lewis C. M. Hamilton S. P. Weissman M. M. (2013). A Mega-Analysis of Genome-wide Association Studies for Major Depressive Disorder. Mol. Psychiatry 18 (4), 497–511. 10.1038/mp.2012.21 22472876 PMC3837431

[B188] Robledo-Menendez A. Vella M. Grandes P. Soria-Gomez E. (2021). Cannabinoid Control of Hippocampal Functions: The where Matters. FEBS J. 10.1111/febs.15907 33977665

[B189] Rodgers R. J. Evans P. M. Murphy A. (2005). Anxiogenic Profile of AM-251, a Selective Cannabinoid CB1 Receptor Antagonist, in Plus-Maze-Naïve and Plus-Maze-Experienced Mice. Behav. Pharmacol. 16 (5–6), 405–413. 10.1097/00008877-200509000-00013 16148445

[B190] Rubino T. Realini N. Castiglioni C. Guidali C. Viganó D. Marras E. (2008). Role in Anxiety Behavior of the Endocannabinoid System in the Prefrontal Cortex. Cereb. Cortex 18 (6), 1292–1301. 10.1093/cercor/bhm161 17921459

[B191] Sánchez M. M. Ladd C. O. Plotsky P. M. (2001). Early Adverse Experience as a Developmental Risk Factor for Later Psychopathology: Evidence from Rodent and Primate Models. Dev. Psychopathol 13 (3), 419–449. 10.1017/s0954579401003029 11523842

[B192] Sanchis-Segura C. Cline B. H. Marsicano G. Lutz B. Spanagel R. (2004). Reduced Sensitivity to Reward in CB1 Knockout Mice. Psychopharmacology (Berl) 176 (2), 223–232. 10.1007/s00213-004-1877-8 15083252

[B193] Santarelli L. Saxe M. Gross C. Surget A. Battaglia F. Dulawa S. (2003). Requirement of Hippocampal Neurogenesis for the Behavioral Effects of Antidepressants. Science 301 (5634), 805–809. 10.1126/science.1083328 12907793

[B194] Sarris J. Sinclair J. Karamacoska D. Davidson M. Firth J. (2020). Medicinal Cannabis for Psychiatric Disorders: A Clinically-Focused Systematic Review. BMC Psychiatry 20 (January), 24. 10.1186/s12888-019-2409-8 31948424 PMC6966847

[B195] Sato K. (2015). Effects of Microglia on Neurogenesis. Glia 63 (8), 1394–1405. 10.1002/glia.22858 26010551 PMC5032973

[B196] Schier A. Ribeiro N. Coutinho D. Machado S. Arias-Carrion O. Crippa J. (2014). Antidepressant-like and Anxiolytic-like Effects of Cannabidiol: A Chemical Compound of Cannabis Sativa. Cnsnddt 13 (6), 953–960. 10.2174/1871527313666140612114838 24923339

[B197] Schloesser R. J. Manji H. K. Martinowich. K. (2009). Suppression of Adult Neurogenesis Leads to an Increased Hypothalamo-Pituitary-Adrenal axis Response. Neuroreport 20 (6), 553–557. 10.1097/WNR.0b013e3283293e59 19322118 PMC2693911

[B198] Scuderi C. Steardo L. Esposito G. (2014). Cannabidiol Promotes Amyloid Precursor Protein Ubiquitination and Reduction of Beta Amyloid Expression in SHSY5YAPP+ Cells through PPARγ Involvement. Phytother Res. 28 (7), 1007–1013. 10.1002/ptr.5095 24288245

[B199] Sexton M. Cuttler C. Finnell J. S. Mischley L. K. (2016). A Cross-Sectional Survey of Medical Cannabis Users: Patterns of Use and Perceived Efficacy. Cannabis Cannabinoid Res. 1 (1), 131–138. 10.1089/can.2016.0007 28861489 PMC5549439

[B200] Shahbazi F. Grandi V. Banerjee A. Trant J. F. (2020). Cannabinoids and Cannabinoid Receptors: The Story So Far. IScience 23 (7), 101301. 10.1016/j.isci.2020.101301 32629422 PMC7339067

[B201] Shao B.-Z. Wei W. Ke P. Xu Z.-Q. Zhou J.-X. Liu C. (2014). Activating Cannabinoid Receptor 2 Alleviates Pathogenesis of Experimental Autoimmune Encephalomyelitis via Activation of Autophagy and Inhibiting NLRP3 Inflammasome. CNS Neurosci. Ther. 20 (12), 1021–1028. 10.1111/cns.12349 25417929 PMC6492996

[B202] Sharir H. Console-Bram L. Mundy C. Popoff S. N. Kapur A. Abood M. E. (2012). The Endocannabinoids Anandamide and Virodhamine Modulate the Activity of the Candidate Cannabinoid Receptor GPR55. J. Neuroimmune Pharmacol. 7 (4), 856–865. 10.1007/s11481-012-9351-6 22454039 PMC3669693

[B203] Sheline Y. I. Wang P. W. Gado M. H. Csernansky J. G. Vannier M. W. (1996). Hippocampal Atrophy in Recurrent Major Depression. Proc. Natl. Acad. Sci. U S A. 93 (9), 3908–3913. 10.1073/pnas.93.9.3908 8632988 PMC39458

[B204] Shen L. Yang Y. Ou T. Key C. C. Tong S. H. Sequeira R. C. (2017). Dietary PUFAs Attenuate NLRP3 Inflammasome Activation via Enhancing Macrophage Autophagy. J. Lipid Res. 58 (9), 1808–1821. 10.1194/jlr.M075879 28729463 PMC5580895

[B205] Shi J. Cai Q. Zhang J. He X. Liu Y. Zhu R. (2017). AM1241 Alleviates MPTP-Induced Parkinson's Disease and Promotes the Regeneration of DA Neurons in PD Mice. Oncotarget 8 (40), 67837–67850. 10.18632/oncotarget.18871 28978077 PMC5620217

[B206] Silva-Cruz A. Carlström M. Ribeiro J. A. Sebastião A. M. (2017). Dual Influence of Endocannabinoids on Long-Term Potentiation of Synaptic Transmission. Front. Pharmacol. 8, 921. 10.3389/fphar.2017.00921 29311928 PMC5742107

[B207] Silverman M. N. PearcePearce B. D. Christine. A. Biron. Biron C. A. Miller A. H. (2005). Immune Modulation of the Hypothalamic-Pituitary-Adrenal (HPA) Axis during Viral Infection. Viral Immunol. 18 (1), 41–78. 10.1089/vim.2005.18.41 15802953 PMC1224723

[B208] Smith S. M. Vale. W. W. (2006). The Role of the Hypothalamic-Pituitary-Adrenal Axis in Neuroendocrine Responses to Stress. Dialogues Clin. Neurosci. 8 (4), 383–395. 17290797 10.31887/DCNS.2006.8.4/ssmith PMC3181830

[B209] Sorrells S. F. Sapolsky R. M. (2007). An Inflammatory Review of Glucocorticoid Actions in the CNS. Brain Behav. Immun. 21 (3), 259–272. 10.1016/j.bbi.2006.11.006 17194565 PMC1997278

[B210] Spector S. Shore P. A. Brodie B. B. (1960). Biochemical and Pharmacological Effects of the Monoamine Oxidase Inhibitors, Iproniazid, 1-Phenyl-2-Hydrazinopropane (JB 516) and 1-Phenyl-3-Hydrazinobutane (JB 835). J. Pharmacol. Exp. Ther. 128 (January), 15–21. 13833205

[B211] Stahl S. M. (1984). Regulation of Neurotransmitter Receptors by Desipramine and Other Antidepressant Drugs: The Neurotransmitter Receptor Hypothesis of Antidepressant Action. J. Clin. Psychiatry 45 (10 Pt 2), 37–45. 6090439

[B212] Starowicz K. Nigam S. Di Marzo V. (2007). Biochemistry and Pharmacology of Endovanilloids. Pharmacol. Ther. 114 (1), 13–33. 10.1016/j.pharmthera.2007.01.005 17349697

[B213] Steiner M. A. Wanisch K. Monory K. Marsicano G. Borroni E. Bächli H. (2008). Impaired Cannabinoid Receptor Type 1 Signaling Interferes with Stress-Coping Behavior in Mice. Pharmacogenomics J. 8 (3), 196–208. 10.1038/sj.tpj.6500466 17684478

[B214] Stella N. (2009). Endocannabinoid Signaling in Microglial Cells. Neuropharmacology 56 Suppl 1 (Suppl. 1), 244–253. 10.1016/j.neuropharm.2008.07.037 18722389 PMC2654419

[B215] Sugama S. Fujita M. Hashimoto M. Conti B. (2007). Stress Induced Morphological Microglial Activation in the Rodent Brain: Involvement of Interleukin-18. Neuroscience 146 (3), 1388–1399. 10.1016/j.neuroscience.2007.02.043 17433555

[B216] Sugiura T. Kishimoto S. Oka S. Gokoh M. (2006). Biochemistry, Pharmacology and Physiology of 2-Arachidonoylglycerol, an Endogenous Cannabinoid Receptor Ligand. Prog. Lipid Res. 45 (5), 405–446. 10.1016/j.plipres.2006.03.003 16678907

[B217] Sugiura T. Kodaka T. Kondo S. Tonegawa T. Nakane S. Kishimoto S. (1997). Inhibition by 2-arachidonoylglycerol, a Novel Type of Possible Neuromodulator, of the Depolarization-Induced Increase in Intracellular Free Calcium in Neuroblastoma X Glioma Hybrid NG108-15 Cells. Biochem. Biophys. Res. Commun. 233 (1), 207–210. 10.1006/bbrc.1997.6425 9144424

[B218] Sugiura T. Kondo S. Kishimoto S. Miyashita T. Nakane S. Kodaka T. (2000). Evidence that 2-Arachidonoylglycerol but Not N-Palmitoylethanolamine or Anandamide Is the Physiological Ligand for the Cannabinoid CB2 Receptor. Comparison of the Agonistic Activities of Various Cannabinoid Receptor Ligands in HL-60 Cells. J. Biol. Chem. 275 (1), 605–612. 10.1074/jbc.275.1.605 10617657

[B219] Sumislawski J. J. Ramikie T. S. Patel. S. (2011). Reversible Gating of Endocannabinoid Plasticity in the Amygdala by Chronic Stress: A Potential Role for Monoacylglycerol Lipase Inhibition in the Prevention of Stress-Induced Behavioral Adaptation. Neuropsychopharmacology 36 (13), 2750–2761. 10.1038/npp.2011.166 21849983 PMC3230498

[B220] Szabo B. Wallmichrath I. Mathonia P. Pfreundtner C. (2000). Cannabinoids Inhibit Excitatory Neurotransmission in the Substantia Nigra Pars Reticulata. Neuroscience 97 (1), 89–97. 10.1016/S0306-4522(00)00036-1 10771342

[B221] Tanaka M. Sackett S. Zhang Y. (2020). Endocannabinoid Modulation of Microglial Phenotypes in Neuropathology. Front. Neurol. 11, 87. 10.3389/fneur.2020.00087 32117037 PMC7033501

[B222] Tanaka M. Yagyu K. Sackett S. Zhang Y. (2019). Anti-Inflammatory Effects by Pharmacological Inhibition or Knockdown of Fatty Acid Amide Hydrolase in BV2 Microglial Cells. Cells 8 (5), 491. 10.3390/cells8050491 PMC6562696 31121907

[B223] Tanasescu R. Constantinescu C. S. (2010). Cannabinoids and the Immune System: An Overview. Immunobiology 215 (8), 588–597. 10.1016/j.imbio.2009.12.005 20153077

[B224] Tang J. Miao H. Jiang B. Chen Q. Tan L. Tao Y. (2017). A Selective CB2R Agonist (JWH133) Restores Neuronal Circuit After Germinal Matrix Hemorrhage In The Preterm Via CX3CR1+ Microglia. Neuropharmacol. 119, 157–169. 10.1016/j.neuropharm.2017.01.027 28153531

[B225] Tao Y. Li L. Jiang B. Feng Z. Yang L. Tang J. (2016). Cannabinoid Receptor-2 Stimulation Suppresses Neuroinflammation by Regulating Microglial M1/M2 Polarization through the CAMP/PKA Pathway in an Experimental GMH Rat Model. Brain Behav. Immun. 58 (November), 118–129. 10.1016/j.bbi.2016.05.020 27261088

[B226] Torres-Berrío Angélica. Issler Orna. Parise Eric. M. Nestler Eric. J. (2019). Unraveling the Epigenetic Landscape of Depression: Focus on Early Life Stress. Dialogues Clin. Neurosci. 21 (4), 341–357. 10.31887/DCNS.2019.21.4/enestler 31949402 PMC6952747

[B227] Trivedi M. H. Rush A. J. Wisniewski S. R. Nierenberg A. A. Warden D. Ritz L. (2006). Evaluation of Outcomes with Citalopram for Depression Using Measurement-Based Care in STAR*D: Implications for Clinical Practice. Am. J. Psychiatry 163 (1), 28–40. 10.1176/appi.ajp.163.1.28 16390886

[B228] Troubat R. Barone P. Leman S. Desmidt T. Cressant A. Atanasova B. (2020). Neuroinflammation and Depression: A Review. Eur. J. Neurosci. 53, 151–171. 10.1111/ejn.14720 32150310

[B229] Turcotte C. Blanchet M. R. Laviolette M. Flamand N. (2016). The CB2 Receptor and its Role as a Regulator of Inflammation. Cell Mol Life Sci 73 (23), 4449–4470. 10.1007/s00018-016-2300-4 27402121 PMC5075023

[B230] Tynan R. J. Naicker S. Hinwood M. Nalivaiko E. Buller K. M. Pow D. V. (2010). Chronic Stress Alters the Density and Morphology of Microglia in a Subset of Stress-Responsive Brain Regions. Brain Behav. Immun. 24 (7), 1058–1068. 10.1016/j.bbi.2010.02.001 20153418

[B231] Uher R. Payne J. L. Pavlova B. Perlis R. H. (2014). Major Depressive Disorder in Dsm-5: Implications for Clinical Practice and Research of Changes from Dsm-Iv. Depress. Anxiety 31 (6), 459–471. 10.1002/da.22217 24272961

[B232] Vahratian A. Blumberg S. J. Terlizzi E. P. Schiller J. S. (2021). Symptoms of Anxiety or Depressive Disorder and Use of Mental Health Care Among Adults during the COVID-19 Pandemic - United States, August 2020-February 2021. MMWR Morb Mortal Wkly Rep. 70 (13), 490–494. 10.15585/mmwr.mm7013e2 33793459 PMC8022876

[B233] Valentino R. J. Van Bockstaele. E. (2008). Convergent Regulation of Locus Coeruleus Activity as an Adaptive Response to Stress. Eur. J. Pharmacol. 583 (2), 194–203. 10.1016/j.ejphar.2007.11.062 18255055 PMC2349983

[B234] Valles-Colomer M. Falony G. Darzi Y. Tigchelaar E. F. Wang J. Tito R. Y. (2019). The Neuroactive Potential of the Human Gut Microbiota in Quality of Life and Depression. Nat. Microbiol. 4 (4), 623–632. 10.1038/s41564-018-0337-x 30718848

[B235] Valverde O. Torrens M. (2012). CB1 Receptor-Deficient Mice as a Model for Depression. Neuroscience 204 (March), 193–206. 10.1016/j.neuroscience.2011.09.031 21964469

[B236] van Laar M. W. Oomen C. J. A. Vercoulen E. Freeman T. P. Hall W. D. (2020). Cannabis and COVID-19: Reasons for Concern. Front. Psychiatry 11. 10.3389/fpsyt.2020.601653 PMC7779403 33408655

[B237] van Praag H. Kempermann G. Gage F. H. (2000). Neural Consequences of Environmental Enrichment. Nat. Rev. Neurosci. 1 (3), 191–198. 10.1038/35044558 11257907

[B238] Van Sickle M. D. Duncan M. Kingsley P. J. Mouihate A. Urbani P. Mackie K. (2005). Identification and Functional Characterization of Brainstem Cannabinoid CB 2 ReceptorsIdentification and Functional Characterization of Brainstem Cannabinoid CB2 Receptors. Science 310 (5746), 329–332. 10.1126/science.1115740 16224028

[B239] Wade M. R. Degroot A. Nomikos G. G. (2006). Cannabinoid CB1 Receptor Antagonism Modulates Plasma Corticosterone in Rodents. Eur. J. Pharmacol. 551 (1–3), 162–167. 10.1016/j.ejphar.2006.08.083 17030030

[B240] Wamsteeker J. I. Kuzmiski J. B. Bains J. S. (2010). Repeated Stress Impairs Endocannabinoid Signaling in the Paraventricular Nucleus of the Hypothalamus. J. Neurosci. 30 (33), 11188–11196. 10.1523/JNEUROSCI.1046-10.2010 20720126 PMC6633493

[B241] Wang M. Hill M. N. Zhang L. Gorzalka B. B. Hillard C. J. Alger B. E. (2012). Acute Restraint Stress Enhances Hippocampal Endocannabinoid Function via Glucocorticoid Receptor Activation. J. Psychopharmacol. 26 (1), 56–70. 10.1177/0269881111409606 21890595 PMC3373303

[B242] Wang W. Sun D. Pan B. Roberts C. J. Sun X. Hillard C. J. (2010). Deficiency in Endocannabinoid Signaling in the Nucleus Accumbens Induced by Chronic Unpredictable Stress. Neuropsychopharmacology 35 (11), 2249–2261. 10.1038/npp.2010.99 20664582 PMC3055309

[B243] Wang Y. Zhang X. (2017). FAAH Inhibition Produces Antidepressant-like Efforts of Mice to Acute Stress via Synaptic Long-Term Depression. Behav. Brain Res. 324 (May), 138–145. 10.1016/j.bbr.2017.01.054 28193523

[B244] Wilson R. I. Nicoll. Roger. A. (2001). “Endogenous Cannabinoids Mediate Retrograde Signalling at Hippocampal Synapses.” Nature 410 (6828), 588–592. 10.1038/35069076 11279497

[B245] Wolf Susanne. A. (2010). “Cannabinoid Receptor CB1 Mediates Baseline and Activity-Induced Survival of New Neurons in Adult Hippocampal Neurogenesis.” Cell Commun. Signaling: CCS 8 (June), 12. 10.1186/1478-811X-8-12 20565726 PMC2898685

[B246] World Health Organization and others (2017). Cannabidiol (CBD) Pre-review Report Agenda Item 5.2. Geneva: Expert Committee on Drug Dependence Thirty-Ninth Meeting.

[B247] World Health Organization (2017). Depression and Other Common Mental Disorders: Global Health Estimates. Geneva. Available at: https://apps.who.int/iris/bitstream/handle/10665/254610/WHO-MSD-MER-2017.2-eng.pdf .

[B248] World Health Organization (2020). Depression in Europe: Facts and Figures.” Depression in Europe: Facts and Figures. Available at: https://www.euro.who.int/en/health-topics/noncommunicable-diseases/mental-health/news/news/2012/10/depression-in-europe/depression-in-europe-facts-and-figures .

[B249] Wu Jiang. Hocevar Mark. Foss Joseph. F. Bie Bihua. Naguib Mohamed. (2017). Activation of CB2 Receptor System Restores Cognitive Capacity and Hippocampal Sox2 Expression in a Transgenic Mouse Model of Alzheimer’s Disease. Eur. J. Pharmacol. 811 (September), 12–20. 10.1016/j.ejphar.2017.05.044 28551012

[B250] Wu Michael. D. Montgomery Sara. L. Escalera Fatima. Rivera. Olschowka John. A. Kerry O’Banion M. (2013). Sustained IL-1β Expression Impairs Adult Hippocampal Neurogenesis Independent of IL-1 Signaling in Nestin+ Neural Precursor Cells. Brain Behav. Immun. 32 (August), 9–18. 10.1016/j.bbi.2013.03.003 23510988 PMC3686979

[B251] Wyrofsky Ryan. R. Reyes Beverly. A. S. Zhang Xiao-Yan. Bhatnagar Seema. Kirby Lynn. G. Van Bockstaele Elisabeth. J. (2019). Endocannabinoids, Stress Signaling, and the Locus Coeruleus-Norepinephrine System. Neurobiol. Stress 11 (November), 100176. 10.1016/j.ynstr.2019.100176 31236436 PMC6582240

[B252] Xu Chen. Chang Tanran. Du Yaqi. Yu Chaohui. Tan Xin. Li Xiangdong. (2019). Pharmacokinetics of Oral and Intravenous Cannabidiol and its Antidepressant-like Effects in Chronic Mild Stress Mouse Model. Environ. Toxicol. Pharmacol. 70 (August), 103202. 10.1016/j.etap.2019.103202 31173966

[B253] Yamada Jun. Jinno Shozo. (2019). Potential Link between Antidepressant-like Effects of Ketamine and Promotion of Adult Neurogenesis in the Ventral Hippocampus of Mice. Neuropharmacology 158 (November), 107710. 10.1016/j.neuropharm.2019.107710 31310776

[B254] Yang Le. Tian Lei. Zhang Zhi. Zhou Xuan. Ji Xiaofang. Liu Fuquan. (2020). Cannabinoid Receptor 1/MiR-30b-5p Axis Governs Macrophage NLRP3 Expression and Inflammasome Activation in Liver Inflammatory Disease. Mol. Ther. - Nucleic Acids 20 (June), 725–738. 10.1016/j.omtn.2020.04.010 32408051 PMC7225604

[B255] Yirmiya Raz. Rimmerman Neta. Ronen Reshef. (2015). Depression as a Microglial Disease. Trends Neurosciences, Neuroimmunology 38 (10), 637–658. 10.1016/j.tins.2015.08.001 26442697

[B256] Yoshida Takayuki. Hashimoto Kouichi. Zimmer Andreas. Maejima Takashi. Araishi Kenji. Kano Masanobu. (2002). The Cannabinoid CB1 Receptor Mediates Retrograde Signals for Depolarization-Induced Suppression of Inhibition in Cerebellar Purkinje Cells. J. Neurosci. 22 (5), 1690–1697. 10.1523/JNEUROSCI.22-05-01690.2002 11880498 PMC6758890

[B257] Zanelati T. V. Biojone C. Moreira F. A. Guimarães F. S. SâmiaJoca R. L. (2010). Antidepressant-like Effects of Cannabidiol in Mice: Possible Involvement of 5-HT1A Receptors. Br. J. Pharmacol. 159 (1), 122–128. 10.1111/j.1476-5381.2009.00521.x 20002102 PMC2823358

[B258] Zanettini Claudio. Panlilio Leigh. V. Aliczki Manó. Goldberg Steven. R. Haller Jozsef. Yasar Sevil. (2011). Effects of Endocannabinoid System Modulation on Cognitive and Emotional Behavior. Front. Behav. Neurosci. 5. 10.3389/fnbeh.2011.00057 PMC3171696 21949506

[B259] Zhang Zhen. Wang Wei. Zhong Peng. Liu Sarah. J. Long Jonathan. Z. Zhao Li. (2015). Blockade of 2-Arachidonoylglycerol Hydrolysis Produces Antidepressant-like Effects and Enhances Adult Hippocampal Neurogenesis and Synaptic Plasticity. Hippocampus 25 (1), 16–26. 10.1002/hipo.22344 25131612 PMC4517601

[B260] Zimmer A. Zimmer A. M. Hohmann A. G. Herkenham M. Tom I. Bonner T. I. (1999). Increased Mortality, Hypoactivity, and Hypoalgesia in Cannabinoid CB1 Receptor Knockout Mice. Proc. Natl. Acad. Sci. 96 (10), 5780. 10.1073/pnas.96.10.5780 10318961 PMC21937

[B261] Zimmermann Tina. Maroso Mattia. Beer Annika. Baddenhausen Sarah. Ludewig Susann. Fan Wenqiang. (2018). Neural Stem Cell Lineage-specific Cannabinoid Type-1 Receptor Regulates Neurogenesis and Plasticity in the Adult Mouse Hippocampus. Cereb. Cortex 28 (12), 4454–4471. 10.1093/cercor/bhy258 30307491 PMC6215469

[B262] Zoppi Silvia. BeatrizNievas G. Pérez. José L. Madrigal M. Manzanares Jorge. Leza Juan. C. (2011). Regulatory Role of Cannabinoid Receptor 1 in Stress-Induced Excitotoxicity and Neuroinflammation. Neuropsychopharmacol. Official Publ. Am. Coll. Neuropsychopharmacol. 36 (4), 805–818. 10.1038/npp.2010.214 PMC3055736 21150911

[B263] Zoppi Silvia. Madrigal José. L. M. BeatrizPérez-Nievas G. Marín-Jiménez Ignacio. Caso Javier. R. Alou Luis. (2012). Endogenous Cannabinoid System Regulates Intestinal Barrier Function *In Vivo* through Cannabinoid Type 1 Receptor Activation. Am. J. PhysiologyGastrointestinal Liver Physiol. 302 (5), G565–G571. 10.1152/ajpgi.00158.2011 22135307

[B264] Zoppi S. Madrigal J. L. Caso J. R. García‐Gutiérrez M. S. Manzanares J. Leza J. C. (2014). Regulatory Role of the Cannabinoid CB2 Receptor in Stress-Induced Neuroinflammation in Mice. Br. J. Pharmacol. 171 (11), 2814–2826. 10.1111/bph.12607 24467609 PMC4243857

[B265] Zuardi Antonio. Waldo. (2006). History of Cannabis as a Medicine: A Review. Braz. J. Psychiatry 28 (2), 153–157. 10.1590/S1516-44462006000200015.c 16810401

