# Supplementary material for: Reviewing the Role of the Endocannabinoid System in the Pathophysiology of Depression
Source: Front Pharmacol. 2021 Dec 6;12:762738. doi: 10.3389/fphar.2021.762738 (PMC8685322; doi:10.3389/fphar.2021.762738)
Supplement: Supplementary file 1 [file Table1.DOCX]

| **Preclinical evidence** | | | |
| --- | --- | --- | --- |
| **Study** | **Species** | **Context** | **Summary of results** |
| Beins (2020) | CB1 KO mice | Lack of CB1, stress and neuroinflammation | Microglial parameters correlated with the severity of the behavioral phenotype. |
| Beyer et al., (2010) | Sprague-Dawley rats | Synthetic CB1 antagonist SR141716 in behavior and neurotransmission and inflammatory biomarkers. | Depressive-like phenotype. Decreased serotonin levels in frontal cortex and hippocampal cell proliferation, survival. Reduced BDNF levels, and increased concentrations of pro-inflammatory cytokines including interferon gamma and TNF alpha. |
| Bridges et al., (2001) | Wistar rats | Synthetic CB1 agonist WIN55,212-2 and neuropathic pain | WIN55,212-2 dose related reversal of all signs of neuropathy. |
| Chen et al., (2018) | Wistar rats | FAAH inhibition and inflammatory-related genes after acute stress. | FAAH inhibition decreases plasma corticosterone at 60 minutes of stress. Attenuation of inflammatory markers expression |
| Fogaça et al., (2018) | C57BL/6 mice | CBD, chronic stress and neurogenesis | Treatment prevents: anxiety-like behavior, decrease in neurogenesis, and dendritic remodeling induced by chronic stress |
| Haller et al., (2002) | CB1 KO mice | Behavioral characterization of CB1-KO mice combined with a cannabinoid antagonist. | Increased anxiety-like behavior reversed by administration of the cannabinoid antagonist. |
| Hill et al., (2008b) | Sprague-Dawley rats | Chronic antidepressant treatment (tranylcypromine and fluoxetine) on cannabinoid CB1 receptor density and endocannabinoid availability. | Reduction of AEA in PFC, hippocampus and hypothalamus and hypothalamus and increased 2-AG in PFC following tranylcypromine administration.  Increased CB1 receptor binding density in PFC and hippocampus. |
| Fogaça et al., (2018) | C57BL/6 mice | CBD, chronic stress and neurogenesis | Treatment prevents: anxiety-like behavior, decrease in neurogenesis, and dendritic remodeling induced by chronic stress |
| Jenniches et al., (2016) | DAGL-α KO mice | Lack of DAGL- α, hence, 2-AG function and anxiety like behavior | Enhanced anxiety, stress and fear responses. |
| Lee & Hill (2013) | Sprague Dawley rats | Restraint stress and CB1 receptor density in PFC, hippocampus and amygdala. | Upregulation of CB1 binding in PFC and amygdala in adolescence |
| Lisboa et al., 2018 | C57BL/6 mice | WIN55,212-2 and stress-induced anxiety like behavior and neuroinflammation. | Reversal of social stress-induced neuroinflammation and anxiety-like symptomatology |
| Litvin et al., 2013 | CB1 KO mice | CB1 in social anxiety and memory. | KOs showed active and passive defensive coping behaviors compared to wild type (WT). CB1KOs and WT receiving an acute dose of CB1 antagonist AM251 displayed anxiety-like behavior during social interaction tests and enhanced levels of social memory. |
| Steiner et al., (2008) | CB1 KO C57BL/6 mice | CB1 receptor in stress coping behaviors | Impaired CB1 receptor function promotes passive stress coping behavior |
| Wang et al., (2010) | CB1 KO C57BL/6 mice | ECS signaling in the nucleus accumbens in chronic unpredictable stress paradigm | Impaired form of endocannabinoid/CB1 mediated plasticity in accumbens. |
| Wang et al.,( 2012) | Sprague Dawley rats | ECS response to acute stress | Delayed increased hippocampal 2-AG content responsible for DSI. |
| Zhang et al., (2015) | C57BL/6 mice | Inhibition of MAGL, chronic stress and neurogenesis | Enhanced adult neurogenesis contributes to anxiolytic like effects of MAGL inhibitor |
| Zoppi et al., (2014) | CB2 KO mice | CB2 receptor and stress-induced neuroinflammation | CB2 absence exacerbated stress-induced neuroinflammatory responses. |

| **Clinical evidence** | | | |
| --- | --- | --- | --- |
| **Study** |  | **Context** | **Summary of results** |
| Domsche et al., (2008) |  | Genetic study of a CB1 polymorphism CNR1rs1049353 | Association to antidepressant resistance |
| Icick et al., (2015) |  | Genetic study of a CB1 polymorphism | Protective influence against MDD |
| Lazary et al., (2019) |  | Polymorphisms of CB2 gene and FAAH and childhood trauma | Both polymorphisms associated to greater sensitivity to childhood trauma. |
| Mitjans et al., (2013) |  | Genetic study of a CB1 polymorphism G allele of rs806371 | Higher presence in patients with MDD. |
| Onaivi et al., 2008 |  | Genetic study of a CB2 polymorphism CNR2rs2501432 | Association between genetic variants of CB2 gene and depressive patients. |
